# Hierarchical Physical‐Cyber Encryption via Metasurface‐Encoded Holographic Keys

**DOI:** 10.1002/advs.76707

**Published:** 2026-07-20

**Authors:** Zhen Liu, Changhong Dai, Wei Zhu, Ruisheng Yang, Bohan Zhang, Lei Zhang, Bobo Du, Shengxiang Wang, Guangwei Hu

**Affiliations:** ^1^ School of Microelectronics Wuhan Textile University Wuhan People's Republic of China; ^2^ State Key Laboratory of New Textile Materials and Advanced Processing Wuhan Textile University Wuhan People's Republic of China; ^3^ School of Electrical and Electronic Engineering Nanyang Technological University Singapore Singapore; ^4^ Key Laboratory for Physical Electronics and Devices of Ministry of Education & Shaanxi Key Laboratory of Information Photonic Technique School of Electronic Science and Engineering Xi'an Jiaotong University Xi'an People's Republic of China

**Keywords:** dynamic codebook, hierarchical encryption, metasurface holography, spin‐decoupling

## Abstract

Physical‐layer encryption based on metasurfaces has emerged as a promising alternative to conventional algorithmic cryptography by embedding security into physical processes. However, most existing metasurface‐based encryption schemes operate within single‐stage or static frameworks, where correct physical illumination directly reveals the hidden information, leaving them vulnerable once the physical key is exposed. Here, we propose a hierarchical physical‐cyber cryptographic framework that synergistically integrates meta‐hologram with dynamic digital protocols to overcome these limitations. The system employs a polarization‐multiplexed metasurface to generate two independent holographic keys through spin‐decoupling wavefront control, establishing a three‐stage cyber‐decryption protocol to combine hardware‐level unclonability with digital algorithmic security. To address the intrinsic noise of physical channels, a robust extraction interface based on amplitude thresholding and grid statistics is further introduced, bridging physical outputs with digital logic and enabling reliable recovery of structured keys from holographic reconstructions under practical interference conditions. Experimental validation in the microwave band confirms stable decryption performance with strong noise resilience, successfully recovering concealed information through sequential physical reconstruction and cryptographic processing. Owing to its inherent frequency‐band universality, the proposed scheme is readily extendable to terahertz and optical frequencies, offering a scalable paradigm for multi‐band secure communication systems. By embedding cryptographic strength in physically unclonable processes, this work not only provides a versatile and intrusion‐resistant framework for next‐generation information protection.

## Abstract 2

A hierarchical physical–cyber encryption framework is demonstrated by integrating a spin‐decoupling metasurface with digital authentication. Two polarization‐dependent holographic keys are extracted from noisy microwave wavefields, combined into a deceptive intermediate ciphertext, and decoded through geometric transformation and a dynamic codebook. This three‐stage strategy bridges physical encryption and deterministic digital logic for robust information protection.

## 1 Introduction

Information security and anti‐counterfeiting technologies form the foundation of modern communication, financial, and defense infrastructures. Conventional cryptography predominantly relies on mathematical complexity‐based algorithms, such as Rivest‐Shamir‐Adleman (RSA) and the Advanced Encryption Standard (AES) [1, 2, 3, 4], whose security is inherently constrained by computational assumptions and therefore increasingly challenged by rapid advances in high‐performance and quantum computing. Optical encryption offers a fundamentally different route by embedding secrecy into physical processes, such as wave propagation, interference, and hardware‐specific responses, thereby shifting protection from purely computational hardness to physically enforced constraints that are difficult to replicate or bypass algorithmically [5, 6, 7, 8, 9]. However, many traditional optical encryption implementations require bulky free‐space components and provide limited controllable degrees of freedom, leading to large footprints and constrained scalability for practical, compact security systems [10, 11, 12, 13].

Metasurfaces, two‐dimensional metamaterials composed by subwavelength planar microstructures, have emerged as a versatile platform for arbitrarily tailoring electromagnetic light waves [14, 15, 16, 17, 18]. Such unprecedented controllability has stimulated extensive exploration based on metasurface‐assisted encryption technologies, ranging from holographic encoding and polarization multiplexing to multi‐channel information storage and processing [19, 20, 21, 22, 23, 24, 25]. Representative works have demonstrated dual‐polarized multiplexed meta holograms, two‐tier manipulation of holographic information, and high‐fidelity multiplexing meta holograms for information display, storage and encryption [26, 27, 28], showing that physical degrees of freedom, such as polarization channels, reconstruction conditions, frequency responses, and propagation directions, can be used to control access to hidden information. These approaches effectively extend encryption from the digital domain to the physical realm by converting keys into diffractive patterns that require precise physical conditions for reconstruction [29, 30, 31], thereby circumventing the intrinsic vulnerabilities of algorithmic encryption through hardware‐enforced protection that is resistant to quantum computational threats [32, 33, 34, 35, 36]. However, most existing metasurface‐based schemes still operate within a single‐stage or static framework, where correct illumination directly reveals the hidden information, leaving the system vulnerable once the physical key or reconstruction rule is exposed [37, 38, 39, 40, 41, 42]. Therefore, developing a hierarchical architecture that incorporates active anti‐counterfeiting layers designed to mislead intruders even after physical access is compromised remains a significant challenge.

In this work, we propose a hierarchical physical‐cyber cryptographic framework that synergistically integrates metasurface holography with dynamic digital protocols. Instead of treating the metasurface output as the final ciphertext, our system employs a spin‐decoupling metasurface to generate two independent holographic keys that serve as intermediate physical primitives for subsequent algorithm processing. By sequentially integrating physical reconstruction, digital matrix extraction, geometric transformation, and final XOR decoding, the framework establishes a highly structured, cyber‐assisted “sandwich” security architecture that combines hardware‐level unclonability with computational flexibility. We design and fabricate two metasurfaces and experimentally demonstrate that they can generate pre‐designed two independent holographic images under the illumination of different circular polarization in the microwave regime. Notably, unlike conventional holography, where smooth focal transitions offer a clear feedback signal for optimization, our quantized extraction interface causes discontinuous bit flips along the propagation path. This mechanism denies attackers a reliable feedback signal and demonstrates that physical‐layer hardware constraints effectively protect the digital decryption process. The proposed noise‐tolerant binary image processing approach, which combines amplitude thresholding with grid‐based statistical analysis, achieves an effective balance between noise suppression and structural integrity, making it well suited for holographic encryption tasks such as key recovery and information reconstruction. Microwave‐band experiments further demonstrate that high‐fidelity holographic reconstruction and reliable binary matrix extraction can be achieved even with a compact metasurface array, highlighting the method's practical viability in resource‐constrained hardware platforms. As a system‐level functional demonstration, this work bridges noisy analog wavefields with deterministic digital logic by combining intermediate holographic key generation, two‐step XOR decoding, and axial‐position‐dependent matrix extraction, providing a hierarchical route for metasurface‐assisted information protection and authentication.

## 2 Generic Strategy for Metasurface‐Encoded Encryption

**FIGURE 1:**
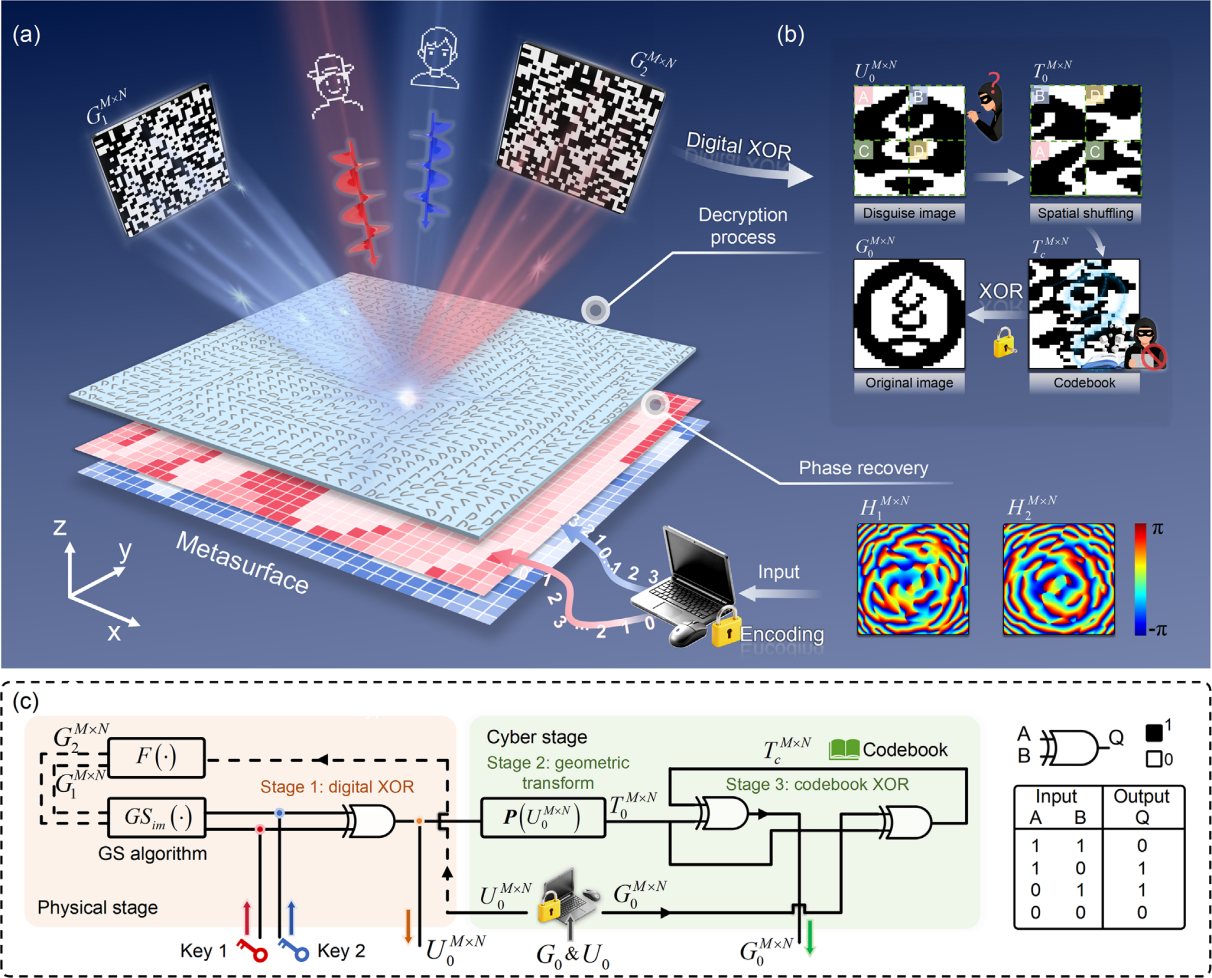
Schematics of the metasurface‐encoded hierarchical encryption and its design workflow. (a) Under circularly polarized light, the metasurface generates two independent holographic keys in the far field, and their digitized XOR yields a deceptive intermediate ciphertext (Stage 1). The authentic image is subsequently recovered via geometric transformation (Stage 2) and dynamic codebook decoding (Stage 3). (b) Flow chart of the design process. (i) For a target image *G*_0_ and its corresponding deceptive disguise image *U*_0_, two binary matrices $G_{1}^{M\times N}$ and $G_{2}^{M\times N}$ are generated via stochastic transformation. (ii) based on the binary key matrices, one can employ the improved GS algorithm to retrieve the corresponding phase distribution $H_{1}^{M\times N}$ and $H_{1}^{M\times N}$. The metasurface is employed to reconstruct the two holographic keys in the far field under different circular polarizations. (iii) The authentic image is finally retrieved through a cyber‐layer decryption process with the dynamic codebook. (c) Comprehensive design and logic block diagram of the hierarchical architecture, integrating the Physical stage and the cyber stage.

We now present the operational principle of the proposed hierarchical physical‐cyber cryptographic framework, ranging from the high‐level cryptographic protocol to its physical implementation based on metasurface holography. As illustrated in Figure 1a, the system integrates a spin‐decoupling metasurface with digital encryption algorithms to establish a multi‐stage encryption and anti‐counterfeiting architecture. Rather than treating the physical output as the final ciphertext, the secret message is first processed at the cyber layer to generate two primary digital keys, which are subsequently encoded into a specially designed metasurface. The metasurface independently manipulates left‐ and right‐circularly polarized (LCP/RCP) waves, enabling the reconstruction of two distinct holographic images at designated diffraction planes under their respective illumination conditions. These reconstructed analog wavefields are sequentially captured by a detector array and discretized into deterministic binary matrices (denoted as Key 1 and Key 2) through a grid‐based statistical extraction interface. A bitwise exclusive OR (XOR) operation is then performed in the digital domain between the two matrices to produce a deceptive visual output rather than the authentic information. In the absence of knowledge of the subsequent decryption protocol, unauthorized interceptors are misled into interpreting this decoy pattern as the recovered message, thereby activating the first layer of active anti‐counterfeiting. Retrieval of the authentic information requires subsequent cyber‐layer operations, where the intermediate decoy undergoes a geometric transform (Stage 2) to diffuse spatial correlations, followed by a final bitwise XOR decoding (Stage 3) with a pre‐shared dynamic codebook library. Therefore, the two XOR operations have distinct functions: the first XOR converts the two physical holographic keys into a visually meaningful decoy, whereas the second XOR functions as the final authentication and information‐recovery step. This cascaded physical–cyber process completes a multi‐stage “sandwich” security architecture, in which physical key generation, logical deception, and dynamic cryptographic authentication collectively ensure robust and intrusion‐resistant information protection. Accordingly, the proposed framework is implemented through a three‐step design procedure (Figure 1b). (i) The original target image is first transformed into a deceptive disguise image, from which two binary key matrices, $G_{1}^{M\times N}$ and $G_{2}^{M\times N}$, are derived via stochastic decomposition. (ii) Based on the obtained binary keys, the corresponding phase‐only distributions, $H_{1}^{M\times N}$ and $H_{2}^{M\times N}$, are retrieved using GS algorithm and subsequently encoded into the metasurface to reconstruct two independent far‐field holographic keys. (iii) A bitwise exclusive OR (XOR) operation is then applied to synthesize the two holographic keys into an intermediate ciphertext $U_{0}^{M\times N}$ via stage 1 decryption. A predefined permutation operation *P*(·) is further performed to generate an intermediate ciphertext, $T_{0}^{M\times N}$, from which the authentic decrypted image is finally recovered via a bitwise XOR operation with the dynamic codebook $T_{c}^{M\times N}$.

First, consider step 1. To encode the authentic secret image *G*_0_ into the physical metasurface, an inverse cryptographic generation process is employed. First, based on the pre‐shared dynamic codebook $T_{C}^{M\times N}$ and the spatial permutation rule *P*, the required intermediate disguise image $U_{0}^{M\times N}$ is back‐calculated from the target information. Subsequently, this calculated $U_{0}^{M\times N}$ is mathematically partitioned into two stochastic binary matrices, designated as $G_{1}^{M\times N}$ and $G_{2}^{M\times N}$ (details in Section S1). This partition is governed by stochastic transformation functions *F*(·), ensuring that the condition $G_{1}^{M\times N}⊕$ $G_{2}^{M\times N}=U_{0}$ is strictly met:

(1) $$\left\{\begin{array}{c} G_{1}^{M\times N}=F_{1}\left(U_{0}\right)\\ G_{2}^{M\times N}=F_{2}\left(U_{0}\right) \end{array}\right.$$

Parallel to this physical encoding, a dynamic codebook ($T_{C}^{M\times N}$) is prepared as a pre‐shared codebook library, in which the corresponding entry is selected in a content‐dependent manner according to the physical decoy and the intended information. Unlike generic stochastic filters, $T_{C}^{M\times N}$ is derived directly as the logical XOR difference between the spatially shuffled physical output and the ground truth information [14, 18]. This derivation ensures that the codebook acts as a perfect complementary matrix, effectively canceling out the deceptive decoy patterns during the final decryption stage to restore the authentic information with high fidelity.

With the binary key matrices defined, the improved Gerchberg‐Saxton (GS) algorithm is next utilized to retrieve their corresponding phase‐only masks, $H_{1}^{M\times N}$ and $H_{2}^{M\times N}$:

(2) $$\left\{\begin{array}{c} H_{1}^{M\times N}=GS_{im}\left(G_{1}^{M\times N}\right)\\ H_{2}^{M\times N}=GS_{im}\left(G_{2}^{M\times N}\right) \end{array}\right.$$

To physically realize these computed holograms, the phase distributions obtained from Equation (2) are integrated into a spin‐decoupling metasurface. Specifically, under LCP illumination, the holographic image of $G_{1}^{M\times N}$ (Key 1) is reconstructed at the diffraction plane *z*_1_. Similarly, under RCP illumination, $G_{2}^{M\times N}$ (Key 2) is reconstructed at the plane *z*_2_.

After physical reconstruction, the two holographic key images are first converted into binary matrices through the matrix‐extraction procedure. A digital bitwise XOR operation is then performed between the two extracted matrices to generate the deceptive ciphertext $U_{0}^{M\times N}$:

(3) $$U_{0}^{M\times N}=G_{1}^{M\times N}⊕G_{2}^{M\times N}$$

Crucially, $U_{0}^{M\times N}$ serves as a visual decoy. If an attacker is unaware of the subsequent steps, they will mistake this coherent image for the final message, thereby activating the first tier of anti‐counterfeiting. This design ensures that successful physical reconstruction and the first‐stage XOR operation remain insufficient for recovering the authentic information. The actual function of $U_{0}^{M\times N}$ is to serve as an operational instruction for stage 2 of decryption. The communicating parties pre‐define its meaning: after obtaining $U_{0}^{M\times N}$, the receiver must first perform a predefined geometric transform on it. This process entails a geometric permutation wherein the image matrix is partitioned into quadrants (e.g., A, B, C, D). An operation is then applied to translate these sub‐matrices to new coordinates, effectively diffusing spatial correlations within the intermediate ciphertext and obtaining an intermediate image $T_{0}^{M\times N}$. This can be formulated as a permutation operation *P*:

(4) $$T_{0}^{M\times N}=P\left(U_{0}^{M\times N}\right)$$

For example, if $U_{0}^{M\times N}$ is partitioned into four sub‐matrices $U_{0}^{M\times N}=\left[\begin{array}{cc} A & B\\ C & D \end{array}\right]$. A potential geometric transform *P* could be to swap the quadrants, resulting in $T_{0}^{M\times N}=\left[\begin{array}{cc} B & D\\ A & C \end{array}\right]$. Subsequently, a second XOR operation is performed between $T_{0}^{M\times N}$ and a Dynamic Codebook ($T_{c}^{M\times N}$) shared by both parties, which enables recovery of the true decrypted image $G_{0}^{M\times N}$.

(5) $$G_{0}^{M\times N}=T_{0}^{M\times N}⊕T_{c}^{M\times N}$$

This final decoding relation also defines the security boundary of the proposed scheme. Under the considered threat model, an attacker may observe the physical decoy but lacks the geometric permutation rule and the corresponding codebook entry; therefore, the authentic information cannot be directly inferred from the decoy alone. For the digit‐based demonstration with ten possible outputs, the blind‐guessing probability is 1/10. For an unconstrained M × N binary message space, the probability of randomly guessing the complete binary matrix scales as 2^−MN^, corresponding to 2^−25^ ≈ 2.98 × 10^−8^ for a 5 × 5 matrix. The complete decryption protocol comprises three sequential stages: initial XOR‐based decryption, followed by a geometric transformation, and culminating in a final XOR decryption phase. This tripartite architecture creates a multi‐stage security framework reminiscent of a sandwich structure (Figure 1c). By integrating the polarization‐multiplexing capabilities of the physical metasurface with dynamic cryptographic protocols from the digital domain, the system achieves substantially enhanced cryptanalytic resistance and demonstrates theoretically‐grounded security robustness.

## 3 Meta‐Atom Design and Experimental Validation of the Encoded Metasurface

**FIGURE 2:**
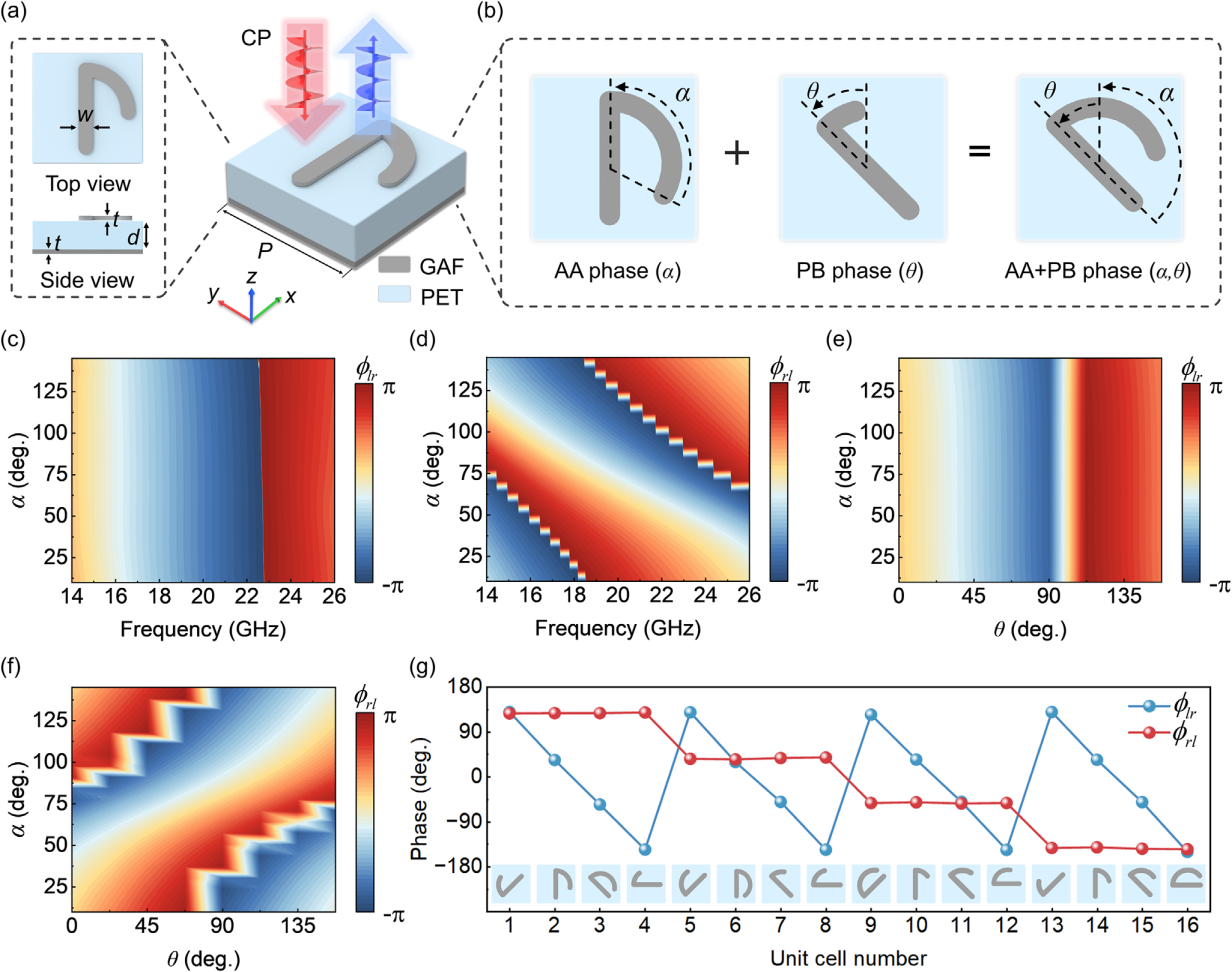
Design and phase‐response characteristics of the spin‐decoupling unit cell. (a) Schematic of the unit cell. (b) Phase modulation principle illustrating the superposition of the Pancharatnam‐Berry (PB) phase and the anisotropic antenna (AA) phase, jointly modulated by the parameters arc arm angle *α* and the rotation angle *θ*. Cross‐polarized reflection phase Φlr (c) and Φlr (d) of the designed unit cell versus frequency and arc arm angle *α*, respectively. Cross‐polarized reflection phase Φlr (e) and Φlr (f) of the designed unit cell versus the rotation angle *θ* and arc arm angle *α*, respectively. (g) Representative spin‐decoupling unit cells selected to cover the full 2π phase range for both polarization channels, together with their corresponding cross‐polarized reflection phases.

To physically realize the proposed dual‐key architecture, the core challenge is to construct a hardware interface that enables independent manipulation of orthogonal polarization channels. To address this, the proposed system relies on a custom‐designed spin‐decoupling metasurface, whose ability to independently control two orthogonal circular polarizations provides the physical mechanism for producing the two unique decryption keys, with the underlying theory and design principles enabling this functionality detailed below [42, 43, 44, 45, 46, 47, 48, 49]. As illustrated in Figure 2a, the proposed meta‐atom is designed as a tri‐layer sandwich structure consisting of a top‐layer patterned Graphene Assembled Film (GAF) and a bottom GAF ground plane separated by a Polyethylene Terephthalate (PET) dielectric substrate with a thickness of d = 2 mm, while the thickness of each GAF layer is 0.05 mm. The physical implementation of the decoupling mechanism relies on the unit cell design illustrated in Figure 2b, which exploits the linear superposition of the Pancharatnam‐Berry (PB) and Analytic Anisotropy (AA) phases [50, 51, 52, 53] dictated by the arc arm angle *α* and the rotation angle *θ* (detailed geometric parameters are provided in Section S2). Owing to its anisotropic geometry, the unit cell exhibits distinct phase responses along two orthogonal principal axes. Let *R*_xx_ and *R*_yy_ denote, respectively, the reflection coefficients of the meta‐atom under illuminations of LP light polarized along two principles axes, we find that the Jones Matrix of the meta‐atom in the LP bases is $J=[\begin{array}{cc} R_{\mathrm{xx}} & 0\\ 0 & R_{\mathrm{yy}} \end{array}]$. The Jones matrix in the circularly polarized basis [32, 33] is given by

(6) $$J_{AA}=\left[\begin{array}{cc} \left|R_{ll}\right|e^{i\frac{\phi_{AA}}{2}} & \left|R_{lr}\right|\\ \left|R_{rl}\right|e^{i\phi_{AA}} & \left|R_{rr}\right|e^{i\frac{\phi_{AA}}{2}} \end{array}\right]$$

When the anisotropic unit mentioned above rotates around its normal by an angle *θ*, its Jones matrix needs to be transformed through a rotation matrix:

(7) $$\begin{array}{c} J_{AA}=\left[\begin{array}{cc} \left|R_{ll}\right|e^{i\frac{\phi_{AA}}{2}}\cos\theta -i\left|R_{lr}\right|\sin\theta & -i\left|R_{ll}\right|e^{i\frac{\phi_{AA}}{2}}\sin\theta +\left|R_{lr}\right|\cos\theta \\ \left|R_{rl}\right|e^{i\phi_{AA}}\cos\theta -i\left|R_{rr}\right|e^{i\frac{\phi_{AA}}{2}}\sin\theta & -i\left|R_{rl}\right|e^{i\frac{\phi_{AA}}{2}}\sin\theta +\left|R_{rr}\right|e^{i\frac{\phi_{AA}}{2}}\cos\theta \end{array}\right] \end{array}$$

To validate this theoretical model, the cross‐polarized reflection phase response was analyzed, as plotted in Figure 2c,d, the measured cross‐polarized reflection phase response clearly demonstrates a distinct asymmetric control mechanism (cross‐polarized reflection amplitudes are provided in Section S3). When the geometric parameter *α* is varied, a specific phase relationship is observed: the phase for the RCP to LCP conversion channel (ϕ_*rl*_) follows the analytical anisotropy phase (ϕ_*AA*_), expressed as ϕ_*rl*_═ ϕ_*AA*_, while the phase for the orthogonal LCP to RCP channel (ϕ_*lr*_) remains constant. Furthermore, the combined influence of *α* and the rotation angle *θ* is visualized in Figure 2e,f. These distinct phase behaviors provide direct confirmation of the decoupling principle, which relies on the linear superposition of the AA and PB phases. This relationship is mathematically formalized as:

(8) $$\left\{\begin{array}{c} \phi_{lr}=\phi_{PB}\left(\theta \right)\\ \phi_{rl}=\phi_{PB}\left(\theta \right)+\phi_{AA}\left(\alpha \right) \end{array}\right.$$

The electric‐field distributions corresponding to the CP responses are provided in Section S4. Based on these governing equations, we constructed a library of 16 spin‐decoupling unit cells, whose reflection phases under LCP and RCP illumination at 19 GHz are presented in Figure 2g. The unit‐cell library collectively exhibits full 2π phase coverage for both polarization channels, thereby validating the design's capability for independent dual‐channel wavefront control.

**FIGURE 3:**
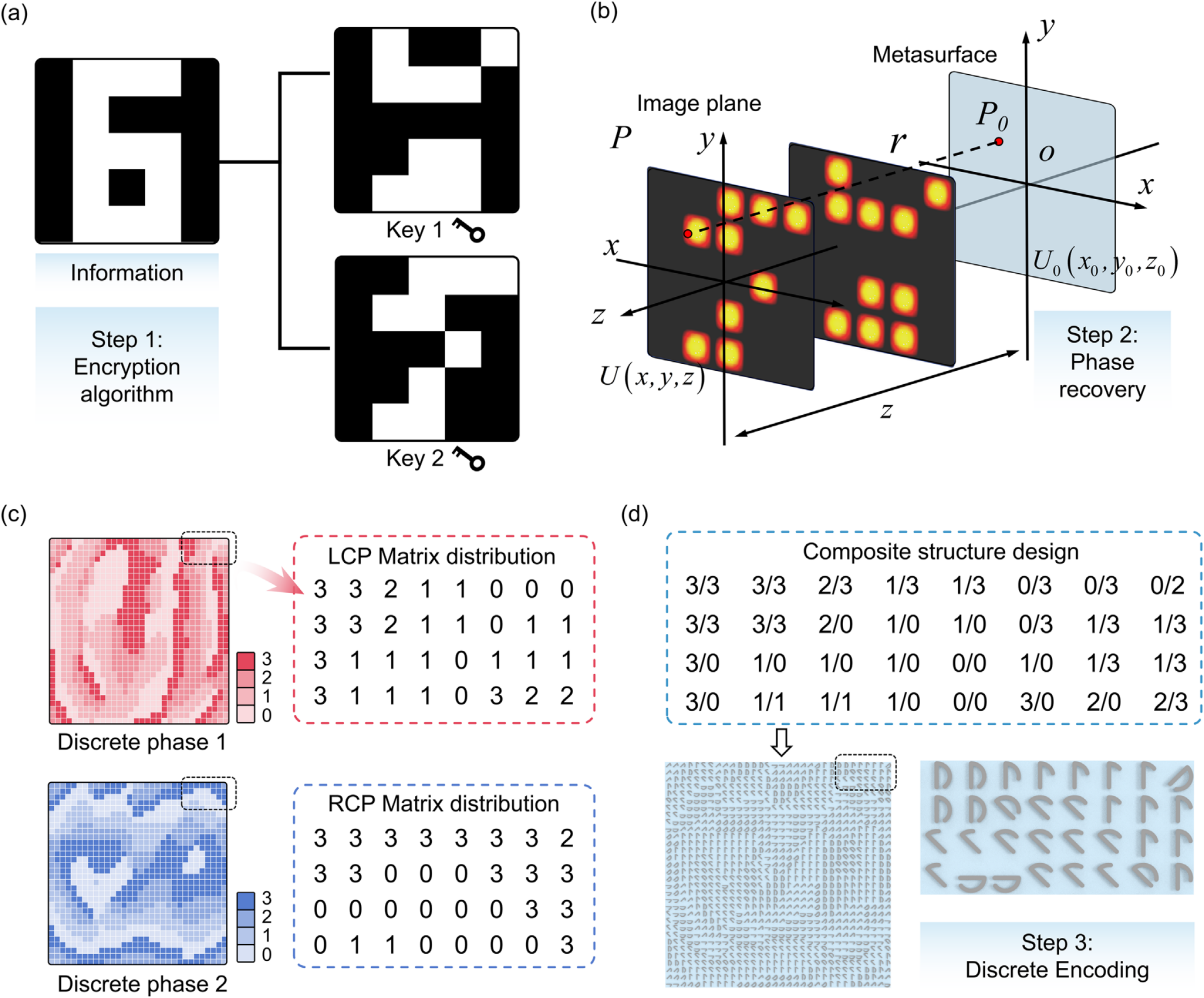
Encoding, design processes, and fabrication of the spin‐decoupling metasurface. (a) Decomposition of the target information (“6”) into two independent key images through digital encryption processing. (b) Discretely encoded phase distributions retrieved by the GS algorithm. (c,d) Encoding process for integrating two independently controlled polarization channels into a single metasurface.

As an illustration of our proposed strategy, we first design and realize two samples corresponding to the encrypted targets “6” and “8”, each of which can generate two holographic keys under LCP and RCP illumination. Figure 3 illustrates the systematic encoding workflow that translates digital information into a physical metasurface representation. The process originates with the target data, exemplified by the digit “6” shown in Figure 3a. Prior to encoding, the image is discretized into a pixelated matrix consisting of uniform blocks. Specifically, the pattern is segmented into a 5 × 5 grid, where all pixels within each block share an identical intensity value, and the entire matrix is binarized to contain only two discrete intensity levels, 0 and 1. In the encryption stage, this binary information is mathematically decomposed into two independent key patterns using the predefined cryptographic algorithm. In the subsequent phase‐recovery stage (Figure 3b), the GS algorithm is employed to retrieve the corresponding phase distributions required for holographic projection of these encoded patterns [54, 55]. Under the Fresnel approximation, the optical field *U*(*x*, *y*, *z*) at the imaging plane can be calculated as:

(9) $$U\left(x,y,z\right)=\frac{e^{jkz}}{j\lambda z}\int \;\;\;\int e^{j\phi \left(x_{0},y_{0}\right)}\cdot e^{j\frac{k}{2z}\left[{\left(x-x_{0}\right)}^{2}+{\left(y-y_{0}\right)}^{2}\right]}dx_{0}dy_{0}$$

where *k* ═ 2π/λ is the wavenumber, (*x*_0_,*y*_0_) and (*x*, *y*) denote coordinates on the metasurface and imaging plane, respectively. The system leverages frequency‐dependent propagation and polarization‐sensitive phase modulation to realize dual‐channel holographic imaging, enabling distinct reconstructed images for LCP illumination at *z*_1_ and RCP illumination at *z*_2_. As shown in Figure 3c, the resulting outputs are two discrete phase matrices, referred to as the LCP and the RCP Matrix distribution. Each cell in these matrices takes a discrete value of (0, 1, 2, 3), corresponding to quantized phase shift 0°, 90°, 180°, and 270°, respectively. In the final encoding stage (Figure 3d), the two phase matrices are mapped onto the physical layout of the metasurface through a composite structural design. At each unit‐cell position, the required pair of phase values extracted from the LCP and RCP matrices uniquely determines the geometric parameters of the corresponding meta‐atom. This encoding strategy ensures that a single metasurface can independently manipulate the phase responses for both polarization channels, thereby enabling the generation of two independent holographic keys within a single integrated device.

**FIGURE 4:**
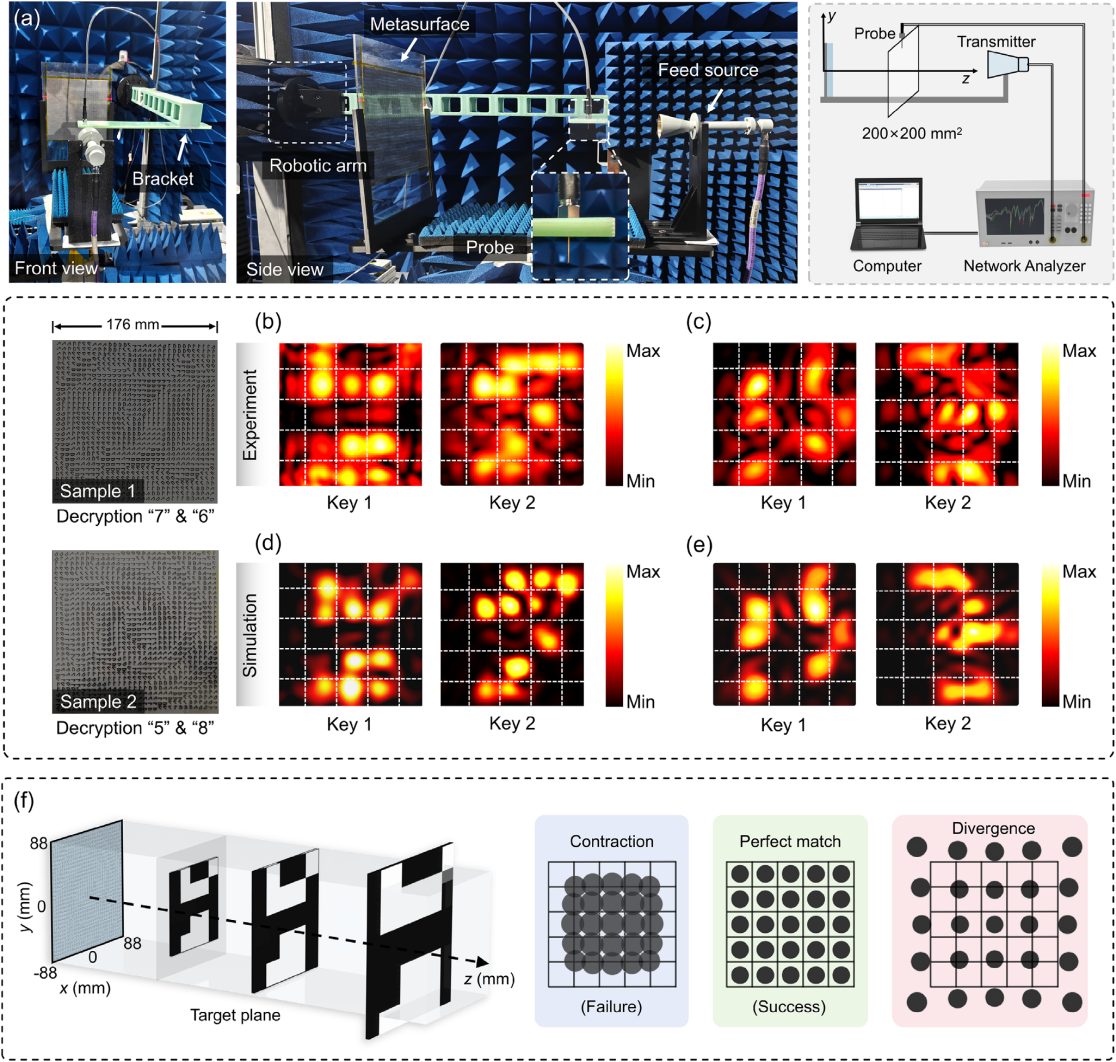
Experimental setup and experimental validation of holographic keys. (a) The microwave near‐field measurement system is used for metasurface characterization. (b, c) Experimentally reconstructed holographic fields for Sample 1 and Sample 2, respectively. (d, e) Corresponding simulated holographic reconstructions for the two samples. (f) Schematic illustration of the contraction and divergence failure mechanisms caused by axial defocusing.

We next conduct near‐field measurements in the microwave Ku‐band to experimentally characterize the holographic images generated by these two samples (fabrication processes of the samples are provided in Section S5). The microwave measurement setup, shown in Figure 4a, is implemented in an anechoic chamber. A CP horn antenna, driven by a vector network analyzer, serves as the excitation source, while the reconstructed holographic field on the image plane is sampled using a waveguide probe mounted on an automated three‐dimensional scanning platform. Figure 4b,c depict the recorded holographic patterns on the imaging plane when the two samples are illuminated under LCP and RCP light, respectively. The generated images show excellent agreement with the numerical simulations in Figure 4d,e. It is worth noting that, as shown in Figure 4f, the geometric fidelity of the retrieved holographic keys is primarily governed by the axial propagation distance Z, which induces both linear geometric scaling and higher‐order diffraction distortion. By deriving a generalized radial displacement model and incorporating the system's spatial tolerance, we establish a strict effective depth‐of‐field within which accurate binary key extraction is guaranteed. In our implementation, for sample 1, the two required holographic keys are generated under LCP illumination at 19 GHz with an imaging distance of 250 mm and under RCP illumination at 19.5 GHz with an imaging distance of 280 mm. For sample 2, the holographic keys are reconstructed under LCP illumination at 18.5 GHz (image plane at 240 mm) and RCP illumination at 18 GHz (image plane at 200 mm). This pronounced longitudinal sensitivity effectively renders the propagation distance Z a physical “positional key”, forming a spatial lock that ensures the valid digital key can only be retrieved under the correct pre‐shared geometric configuration (detailed derivations are provided in Section S6).

**FIGURE 5:**
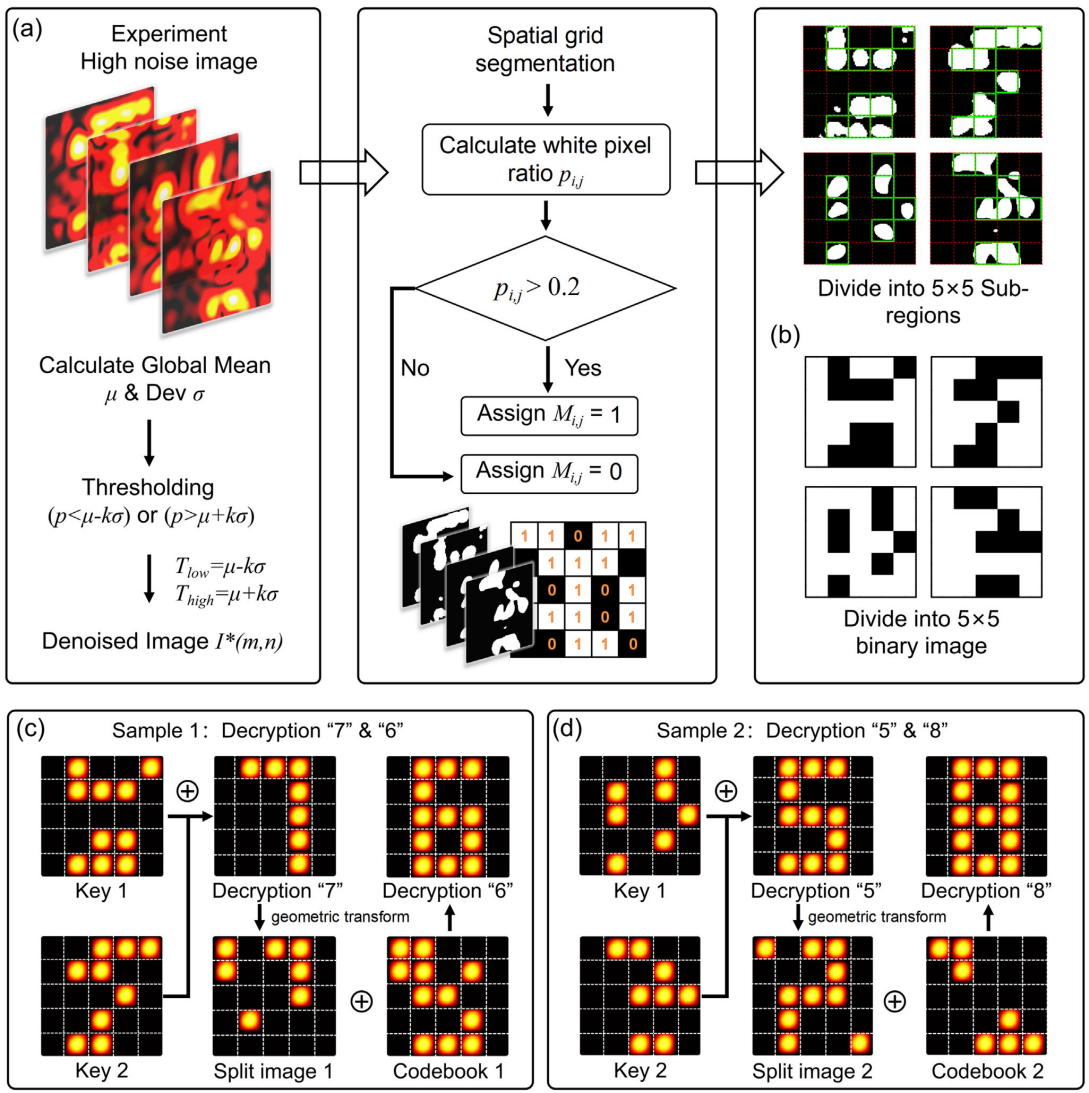
Binary matrix extraction workflow and hierarchical physical–cyber decryption process. (a) Schematic illustration of the binary image denoising and matrix extraction procedure that bridges the noisy physical measurements and the digital cryptographic processing. (b) Binary matrices extracted from experimentally reconstructed holographic images through the denoising and matrix extraction workflow. (c, d) Stepwise decryption processes for sample 1 and sample 2, respectively, illustrating the evolution from the deceptive stage 1 ciphertext to the authentic information through geometric transformation and XOR decryption assisted by the dynamic codebook.

Although the reconstructed raw images exhibit characteristic physical noise and clutter, a common challenge in near‐field holographic measurements, the proposed hierarchical framework remains robust. To mitigate such interferences, as shown in Figure 5a, a block‐based matrix extraction strategy is established to bridge the analog physical domain with the digital cryptographic protocol. Owing to unavoidable background noise arising from speckle effect, alignment tolerances, and environmental disturbances, direct visual inspection of experimental holographic reconstruction is insufficient for reliable key retrieval. Therefore, the proposed algorithm goes beyond simple image enhancement by serving as a robust analog‐to‐digital converter that efficiently translates the noisy physical field intensity into a deterministic 5 × 5 binary logic matrix. The extraction procedure begins with amplitude‐based denoising, in which pixels whose amplitude falls outside the threshold range defined by {*T_l_* ═ μ − *k*σ, *T_h_* ═ μ + *k*σ} are replaced using the mean of their valid neighboring pixels. Here, *µ* represents the global mean amplitude of the reconstructed image, *σ* denotes the corresponding standard deviation of the pixel amplitudes, and *k* is a tunable scaling factor controlling the noise rejection strength. Following denoising, the image is divided into a uniform 5 × 5 grid, and each block *B*_*i*,*j*_ is assigned a binary value according to:

(10) $$M_{i,j}=\left\{\begin{array}{c} 1,\left(\frac{\sum_{\left(x,y\right)\in B_{i,j}}I^{*}\left(x,y\right)}{\left|B_{i,j}\right|}>p\right)\\ 0,\mathrm{otherwise} \end{array}\right.$$

where *p* is a predefined ratio threshold (details on thresholding and scalability in Section S9). This structured processing pipeline enables reliable conversion of noisy holographic inputs into well‐defined binary matrices, facilitating subsequent cryptographic processing and accurate information reconstruction. Figure 5b depicts the extracted matrix results from the holographic images shown in Figure 4b, confirming that the security system does not require perfect, noise‐free physical conditions to function, but rather relies on the synergistic interplay between physical irreversibility and algorithmic error correction to ensure data integrity (multi‐frequency performances are provided in Section S7). As shown in Figure 5c,d, the decryption processes for the two samples are systematically demonstrated. In both cases, the decryption process begins with an encrypted image characterized by sparsely distributed bright spots, undergoes reconfiguration via a predefined geometric transformation to form an intermediate pattern, and culminates in the final recovery of the true information through a bitwise XOR operation between this transformed image and its corresponding dynamic codebook, which is defined by Equation (5). Notably, the initial decryption stage yielded misleading numerical patterns, specifically displaying “7” for sample 1 and “5” for sample 2, which function as deceptive keys. The authentic information (“6” and “8”, respectively) is exclusively recovered after the final XOR operation with the dynamic codebook (more details can be found in Section S8), thereby validating the effectiveness of the complete three‐stage cyber decryption protocol. From a cryptographic perspective, this mechanism fundamentally differs from traditional static‐key schemes: the codebook, constructed as the inverse complement of the physical decoy relative to the original image, functions as a selected entry from the pre‐shared dynamic codebook library, preventing direct recovery of the authentic information from the intercepted physical patterns alone without the geometric permutation rule and the corresponding codebook entry. Regarding the information capacity, the 5 × 5 pixel matrix employed in this proof‐of‐concept demonstration is chosen to match the resolution limits of the microwave scanning systems and to clearly visualize the binary extraction logic, rather than a fundamental limitation. In higher‐frequency regimes, metasurfaces have demonstrated the capability to reconstruct micrometer‐scale pixels with high efficiencies, indicating that the proposed sandwich security architecture and the dual‐channel spin‐decoupling mechanism are theoretically compatible with high‐resolution implementations. With appropriate aperture‐field engineering or inverse design strategies to suppress pixel crosstalk, the current 5 × 5 grid can, in principle, be scaled to megapixel arrays without altering the underlying cryptographic protocol.

## 4 Conclusions

This work presents a secure communication framework that transcends conventional algorithm‐encryption methods by tightly integrating physical‐layer properties with cryptographic protocols. By employing a spin‐decoupling metasurface to generate independent holographic keys, we establish a three‐stage cyber decryption protocol are tightly cascaded to form a robust information protection architecture. Experimental results confirm that dual‐channel holographic projection can be reliably achieved, validating the effectiveness of the proposed physical‐cyber integration. To ensure robustness under realistic conditions, a noise‐tolerant binary image processing strategy based on amplitude thresholding and grid statistics is introduced. Experiments in the microwave band demonstrate that high‐fidelity holographic reconstruction and accurate binary matrix extraction are attainable even with compact metasurface arrays, confirming its practical utility in resource‐constrained hardware environments. Beyond experimental validation, this study provides a proof‐of‐concept system demonstration of hierarchical physical–cyber encryption, where established metasurface holography and digital logic operations are coupled through intermediate key generation, two‐step XOR decoding, and axial‐position‐dependent matrix extraction. The proposed framework offers a cost‐effective solution that requires only a single metasurface while maintaining high security standards. As such, it provides a versatile foundation for developing secure communication systems that are inherently resistant to evolving computational threats, thus opening new possibilities for future information protection.

## 5 Experimental Section

All experiments are conducted in the microwave Ku‐band, which serves two primary purposes. First, it enables precise full‐vector electromagnetic field measurements to rigorously validate the phase‐addressable decoupling mechanism. Second, it demonstrates the direct applicability of the physical‐cyber security framework to microwave systems, which are fundamental to modern wireless communication and radar technologies.

### 5.1 Simulation Model

Numerical simulations for metasurface design and performance verification are conducted using the commercial software CST Studio Suite. The simulation workflow is performed at two levels: the unit cell analysis and the full‐wave metasurface simulation. For the unit‐cell analysis, a unit cell is modeled with Floquet periodic boundary conditions along the *x*‐ and *y*‐directions, and a frequency‐domain solver is employed to sweep key parameters, including the arc arm angle *α* and the rotation angle *θ*, across the Ku‐band. The corresponding S‐parameters are extracted to characterize the cross‐polarized reflection amplitudes and phase under LCP and RCP illumination. Subsequently, the full‐wave simulations are performed for the complete metasurface structure comprising a 32 × 32 unit‐cell array, with open (add space) boundary conditions and CP plane‐wave excitation. Electric‐field monitors are positioned at the corresponding target planes to capture the reconstructed intensity patterns, thereby verifying the independent holographic image formation and the performance of the physical encryption scheme.

### 5.2 Fabrication

The metasurface samples were fabricated using a multi‐step laser micromachining and assembly process to accurately translate the simulated phase profiles into physical structures. Metasurface samples were fabricated via laser micromachining. A GAF is laminated onto a flexible PET substrate, followed by precise laser ablation to engrave the optimized unit cell geometries. The periodic patterns were then precisely performed using a UV picosecond precision laser system with a standard wavelength of 355 nm and an F100 mm field lens, operating at a repetition rate of 700 kHz, a pulse width of 8 ps, and an average power of 7.5 W (single‐pulse energy of 10 µJ). Each pattern is scanned 50 times at a speed of 1000 mm/s to ensure clean edges without damaging the substrate. Following the removal of excess material, the bilayer architecture was assembled by incorporating a 1.9 mm PET spacer.

### 5.3 Near‐Field Measurement

The near‐field measurement system is established inside a microwave anechoic chamber to suppress external electromagnetic interference. The measurement setup employs a vector network analyzer (VNA) as the core instrumentation platform for generating and receiving Ku‐band microwave signals, while a CP horn antenna connected to the VNA served as the feed source to illuminate the metasurface samples with designated LCP/RCP plane waves at their specific operational frequencies. To characterize the reconstructed holographic field distributions, a rectangular waveguide probe mounted on a computer‐controlled three‐axis automated scanning platform is employed to perform systematic raster scans across predefined image planes, capturing complex electric field data with high spatial resolution through coordinated amplitude and phase measurements. Following data acquisition, the near‐field information is processed to derive intensity distributions corresponding to the projected holographic keys, with comprehensive measurements conducted for each sample under LCP and RCP illumination conditions at their respective operating frequencies and specified imaging distances to fully validate the metasurface's decryption capabilities.

### 5.4 Decryption Processing Experiment

The decryption process is experimentally validated using a combined physical measurement and computational analysis workflow. Following the holographic reconstruction of two distinct key patterns under specific polarization and frequency conditions, the captured images are automatically processed using a customized image recognition algorithm. The algorithm employed block‐based matrix extraction with adaptive thresholding and morphological operations to convert the grayscale holographic images into high‐fidelity binary images. The decryption protocol is experimentally validated by processing the captured holograms. An automated algorithm performs sequential XOR operations between the extracted binary matrices and the dynamic codebook, successfully recovering the original information.

## Author Contributions

**Changhong Dai**: conceptualization, methodology, visualization, Writing – review and editing. **Zhen Liu**: conceptualization, methodology, validation, writing – original draft. **Guangwei Hu**: conceptualization, investigation, methodology, supervision, resources, validation, writing – review and editing. **Bohan Zhang**: methodology, visualization, software, data curation, writing – review and editing. **Wei Zhu**: methodology, software, data curation, formal analysis, writing – review and editing. **Lei Zhang**: methodology, formal analysis, validation, writing – review and editing. **Bobo Du**: validation, formal analysis, project administration, writing – review and editing. **Shengxiang Wang**: writing – review and editing, methodology, visualization. **Ruisheng Yang**: software, visualization, project administration, writing – review and editing.

## Conflicts of Interest

The authors declare no conflicts of interest.

## Supporting information




**Supporting File**: advs76707‐sup‐0001‐SuppMat.docx.

## Data Availability

The data that support the findings of this study are available from the corresponding author upon reasonable request.
