# Supplementary material for: Hierarchical Physical‐Cyber Encryption via Metasurface‐Encoded Holographic Keys
Source: Adv Sci (Weinh). 2026 Jul 20:e76707. Online ahead of print. doi: 10.1002/advs.76707 (PMC13384417; doi:10.1002/advs.76707)
Supplement: Supplementary file 1 — Supporting File: advs76707‐sup‐0001‐SuppMat.docx. [file ADVS-9999-e76707-s001.docx]

Supporting Information

**Hierarchical Physical-Cyber Encryption via Metasurface-Encoded Holographic Keys**

*Zhen Liu^1, 2#^, Changhong Dai^3^, Wei Zhu^1, 2*^, Ruisheng Yang^4*^, Bohan Zhang^1, 2^, Lei Zhang^4^, Bobo Du^4^, Shengxiang Wang^1, 2*^, Guangwei Hu^3*^*

^1^ *School of Microelectronics, Wuhan Textile University, Wuhan, People's Republic of China*

^2^ *State Key Laboratory of New Textile Materials and Advanced Processing, Wuhan Textile University, Wuhan, People's Republic of China*

^3^ *School of Electrical and Electronic Engineering, Nanyang Technological University, Singapore, Singapore*

^4^ *Key Laboratory for Physical Electronics and Devices of Ministry of Education & Shaanxi Key Laboratory of Information Photonic Technique, School of Electronic Science and Engineering, Xi'an Jiaotong University, Xi'an, People's Republic of China*

**Section 1. Encryption process**

**Section 2. Unit cell parameters**

**Section 3. Cross-polarized reflection amplitude**

**Section 4. Electric field distributions of LCP and RCP responses**

**Section 5. Fabrication process of samples**

**Section 6. Quantitative analysis of longitudinal tolerance and spatial locking mechanism**

**Section 7. Multi frequency performance
Section 8. Visualization of the dynamic codebook library.**

**Section 9. Evaluation of the threshold and demonstration of capacity scalability**

**Section 1. Encryption process**


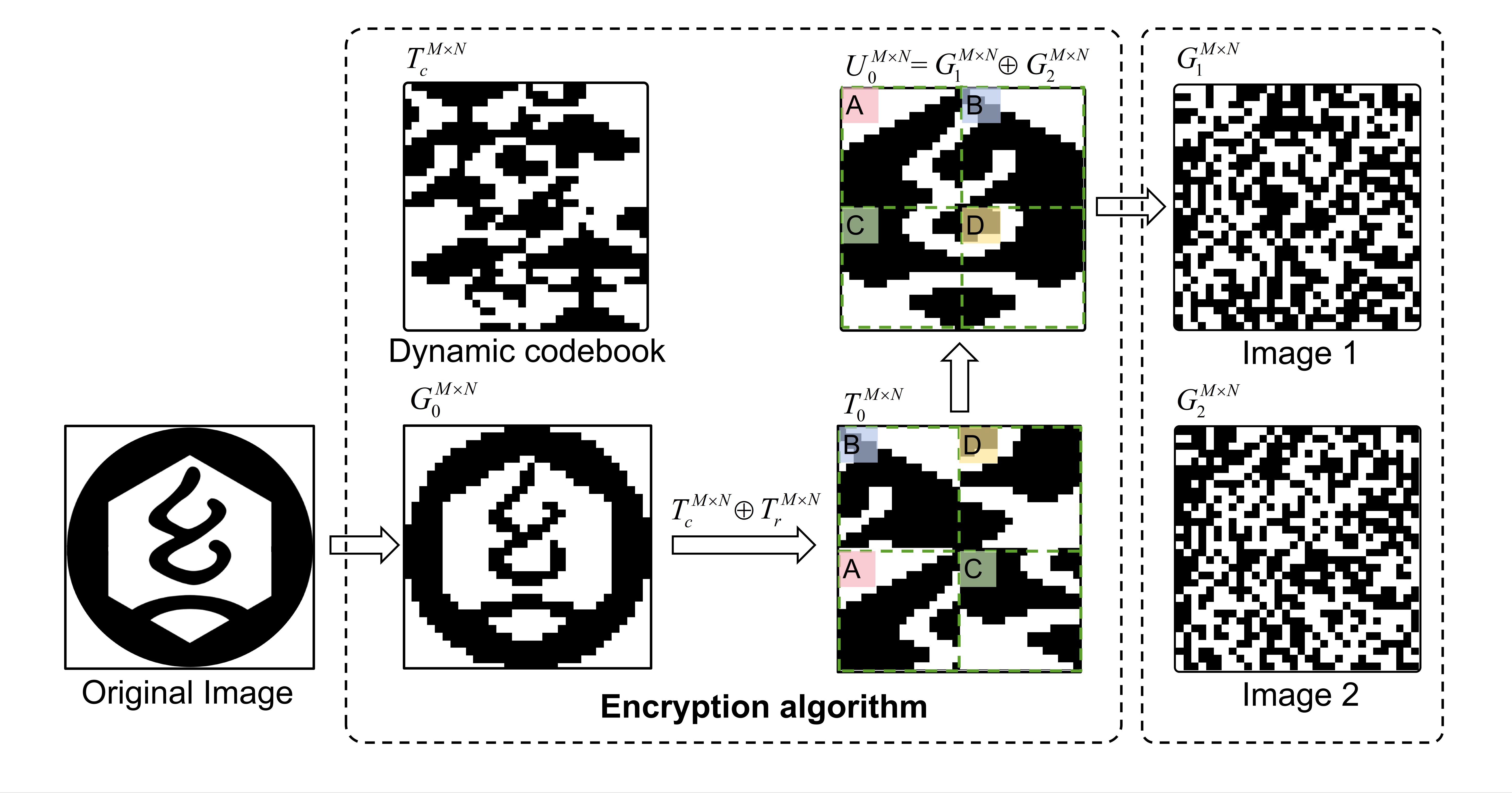


**Figure S1.** The encryption process of splitting the original image into Image 1 and Image 2.

As shown in **Figure S1**, the original image is first converted into a binary image $T_{r}^{M\times N}$ by computational processing. $T_{r}^{M\times N}$ is then XORed with a dynamic codebook $T_{c}^{M\times N}$ to obtain an intermediate image $T_{0}^{M\times N}$. Subsequently, $T_{0}^{M\times N}$ undergoes regional transformation and segmentation, producing image $U_{0}^{M\times N}$. Finally, $U_{0}^{M\times N}$ is accurately segmented into image 1 and image 2 using a random inverse-XOR splitting procedure. The key to this algorithm lies in its "reverse" design thinking: it does not simply divide the original image, but introduces a random sequence so that image 1 and image 2 do not contain any readable information when they exist separately, and there is no obvious statistical correlation between the two.

In summary, through multi-stage operations of "binarization-dynamic encryption-geometric transformation-random segmentation," this preprocessing pipeline transforms a simple digital secret into two seemingly random and mutually independent images.

The detailed step-by-step procedure for the "Random Inverse XOR Splitting" algorithm is presented in Algorithm 1.

**Algorithm 1: Random inverse-XOR splitting for Holographic Key Generation**

**Input:**

**U0 : Binary matrix of the intermediate decoy image (Size M x N)**

**Seed : Optional integer seed for reproducibility**

**Output:**

**G1 : Binary matrix for Key 1 (LCP Channel)**

**G2 : Binary matrix for Key 2 (RCP Channel)**

**Procedure:**

**1. Initialize matrices G1 and G2 with size M x N**

**2. Set Random_Seed(Seed) // Ensure cryptographic randomness**

**3. // Iterate through every pixel position**

**For i from 1 to M do:**

**For j from 1 to N do:**

**// Step A: Generate a random bit r from {0, 1}**

**r = Random_Integer(0, 1)**

**// Step B: Assign the random bit to G1**

**G1[i, j] = r**

**// Step C: Calculate G2 using Inverse XOR logic**

**// If U0[i,j] is 1, G2 must be opposite to G1**

**// If U0[i,j] is 0, G2 must be equal to G1**

**G2[i, j] = U0[i, j] XOR r**

**End For**

**End For**

1. **Return G1, G2**

To ensure the reproducibility of the proposed hierarchical physical-cyber encryption scheme, we provide the rigorous mathematical formulation and the corresponding pseudocode for the key generation process. The core objective of this algorithm is to decompose the target intermediate decoy image ($U_{0}$) into two statistically independent key matrices, which serve as the input for the metasurface design.

Let $U_{0}$ be the binary intermediate decoy image matrix of size $M\times N$, where $U_{0}\left(i,j\right)\in\left\{\left.0,1\right\}\right.$. The decomposition process employs a random splitting protocol based on the exclusive-OR (XOR) operation properties.

We introduce a cryptographic-grade random binary matrix *R* of the same dimension$M\times N$, generated via a secure random number generator (RNG). The elements of *R* are uniformly distributed:

$P\left(R\left(i,j\right)=0\right)=P\left(R\left(i,j\right)=1\right)=0.5$ (S1)

The two holographic key matrices, $G_{1}$ and $G_{2}$, are defined as follows:

Key 1 assignment: The first key is directly assigned the values of the random matrix *R*.

$G_{1}\left(i,j\right)=R\left(i,j\right)$ (S2)

Key 2 calculation: The second key is derived by performing a bitwise XOR operation between the target decoy image $U_{0}$ and the random matrix $R$.

$G_{2}\left(i,j\right)=U_{0}\left(i,j\right)\oplus R\left(i,j\right)$ (S3)

Since $G_{1}$ is purely random and $G_{2}$ is obtained by bitwise XORing the target decoy image $U_{0}$ with the same random matrix both $G_{1}$ and $G_{2}$ individually appear as random noise, so no information about $U_{0}$ can be retrieved from either key alone and they must be physically or digitally combined to reveal the decoy image.

**Section 2.** **Unit cell parameters**


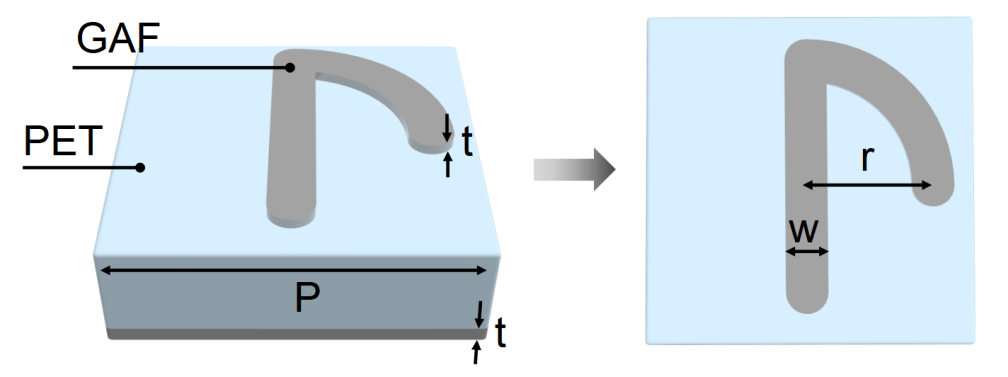


**Figure S2.** Unit cell parameters and material properties.

As shown in **Figure S2**, the unit cell exhibits a periodicity of P = 5.5 mm with a metallic line width of w = 0.7 mm and a structural radius of r = 1.75 mm. The configuration incorporates a polyethylene terephthalate (PET) dielectric substrate with thickness d = 2.0 mm, characterized by a relative permittivity of ε_r_ = 3.2. The graphene assembled film (GAF) demonstrates a thickness of t = 0.05 mm with an electrical conductivity of σ = 1.1×10^6^ S/m.


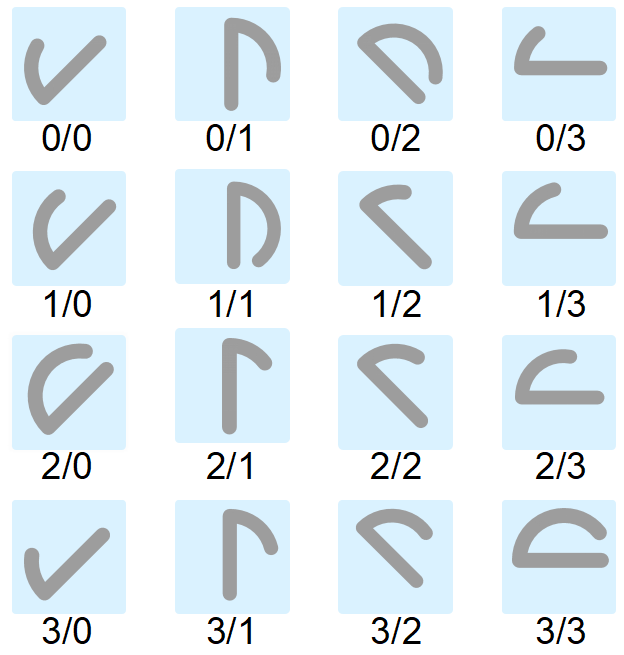


**Figure S3.** Matrix organization of the 16-unit-cell library.

As shown in **Figure S3**, the 16 decoupling unit cells exhibit full 360° cross-polarized phase coverage under LCP and RCP illumination. The core of the polarization-decoupling metasurface is a library of 16 distinct unit cells, systematically organized in a 4x4 matrix format. Each cell is uniquely identified by a row/column index, ranging from 0/0 to 3/3.


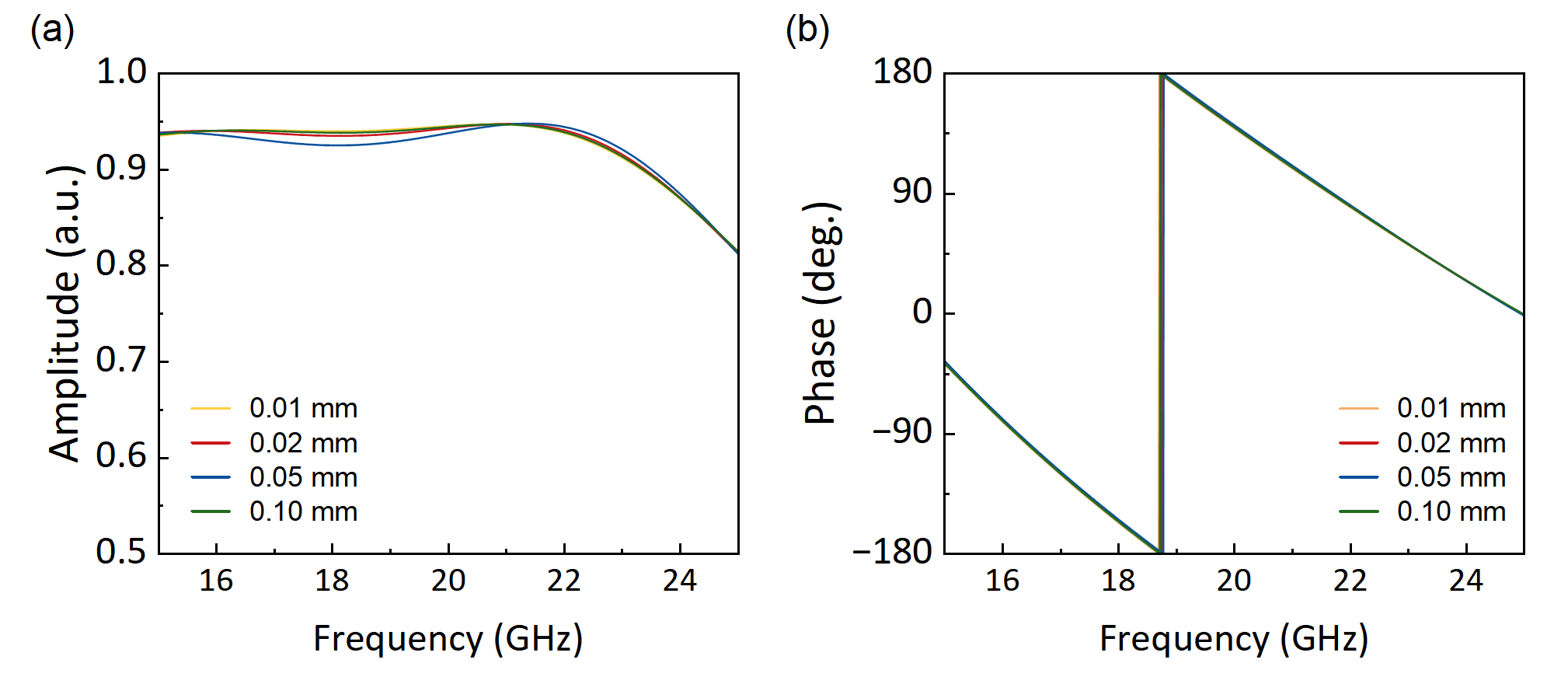


**Figure S4.** Validation of the effective thickness model. Simulated (a) amplitude and (b) phase.

To validate the thickness configuration, a parameter sweep was conducted for thicknesses ranging from 0.01 mm to 0.10 mm. As illustrated in **Figure S4**, the transmission amplitude and phase responses exhibit negligible variations. Because these thicknesses are deeply subwavelength at microwave frequencies, they do not significantly perturb the overall electromagnetic resonance.

**Table S1.** 2-bit encoding unit cell parameters for phase control.

| *N_lr_*/*N_rl_* | 0/0 | 0/1 | 0/2 | 0/3 |
| --- | --- | --- | --- | --- |
| (*α*, *θ*) | (76, 135) | (100, 0) | (142, 45) | (53, 90) |
|  | 1/0 | 1/1 | 1/2 | 1/3 |
|  | (100, 135) | (142, 0) | (53, 45) | (76, 90) |
|  | 2/0 | 2/1 | 2/2 | 2/3 |
|  | (142, 135) | (53, 0) | (76, 45) | (100, 90) |
|  | 3/0 | 3/1 | 3/2 | 3/3 |
|  | (53, 135) | (76, 0) | (100, 45) | (142, 90) |

Of particular importance to the realization of the polarization-decoupling functionality is the precise design of the meta-atoms, as conceptually summarized in a theoretical parameter **Table S1** for 2-bit phase encoding. This table essentially defines the mapping between the geometric parameters of the unit cells—specifically the arc arm angle *α* and the rotation angle *θ*—and their corresponding cross-polarized reflection phases, which are critical for independent wavefront control. Within this framework, the parameters *N_lr_* and *N_rl_* represent the independent phase-encoding states for the two orthogonal circular polarization conversion channels: *N_lr_* corresponds to the phase shift for the LCP-to-RCP conversion path, while *N_rl_* corresponds to that for the RCP-to-LCP path. The combination of (*α*, *θ*) serves as the fundamental 2-bit encoding scheme, enabling the creation of 16 distinct unit cells that provide the necessary 0°, 90°, 180°, and 270° phase shifts to achieve full 360° phase coverage for both polarization states.

**Section 3. Cross-polarized reflection amplitude**


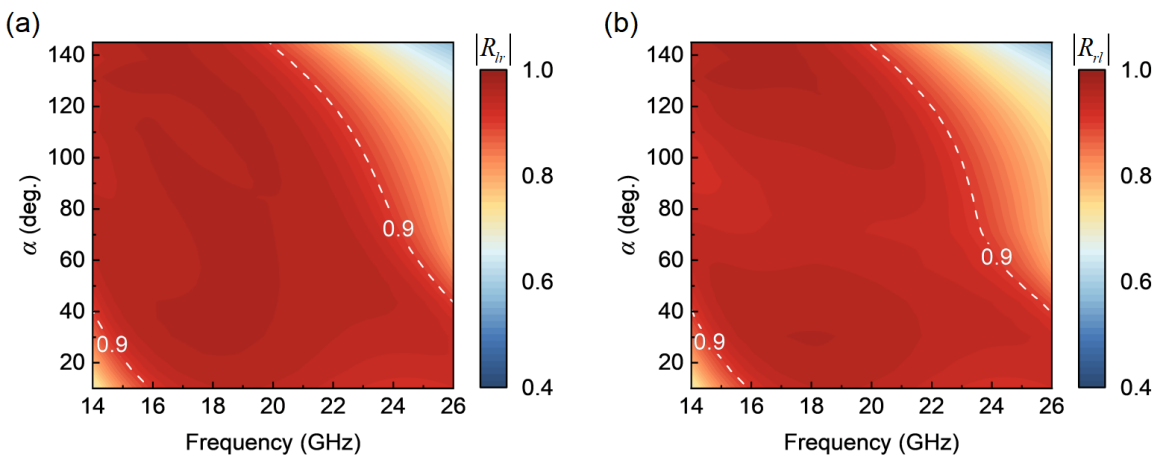


Figure S5. Cross-polarized reflection amplitude (a) and (b) ($\left|R_{lr}\right|$ and $\left|R_{rl}\right|$ ) of the designed unit cell versus frequency and arc arm angle *α*.

As shown in Figure S5, the cross-polarized reflection amplitudes $\left|R_{lr}\right|$ and $\left|R_{rl}\right|$ of the designed unit cell are plotted as functions of frequency and the arc arm angle α. The distinct curved high-response regions in both subfigures demonstrate a broad operating bandwidth covering 15–25 GHz, which corresponds to a relative bandwidth exceeding 40%. Within this range, the reflection amplitudes for both polarization channels remain consistently above 0.9, indicating highly efficient polarization conversion performance across a wide spectrum.

**Section 4. Electric field distributions of LCP and RCP responses**


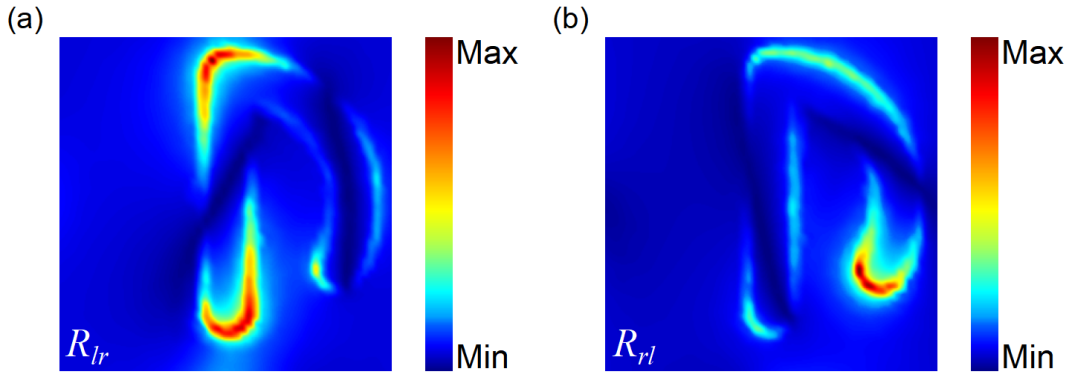


Figure S6. Electric field distributions of the cross-polarized reflections $R_{lr}$ (a) and $R_{rl}$ (b).

As shown in **Figure S6**, the electric field distributions of the cross-polarized reflections and provide an intuitive visualization of the independent control mechanism for LCP and RCP waves. Under LCP excitation, the electric field is strongly localized around the central linear segment due to resonance, while under RCP excitation, the field concentrates along the curved arms. This clear spatial separation of resonant responses enables spin-decoupled wavefront manipulation: the linear segment imparts a spin-dependent PB phase through structural rotation, whereas the curved arms introduce a geometric phase via arc-length modulation. Together, this configuration allows independent and simultaneous wavefront control for orthogonal circular polarizations.

**Section 5. Fabrication process of samples**


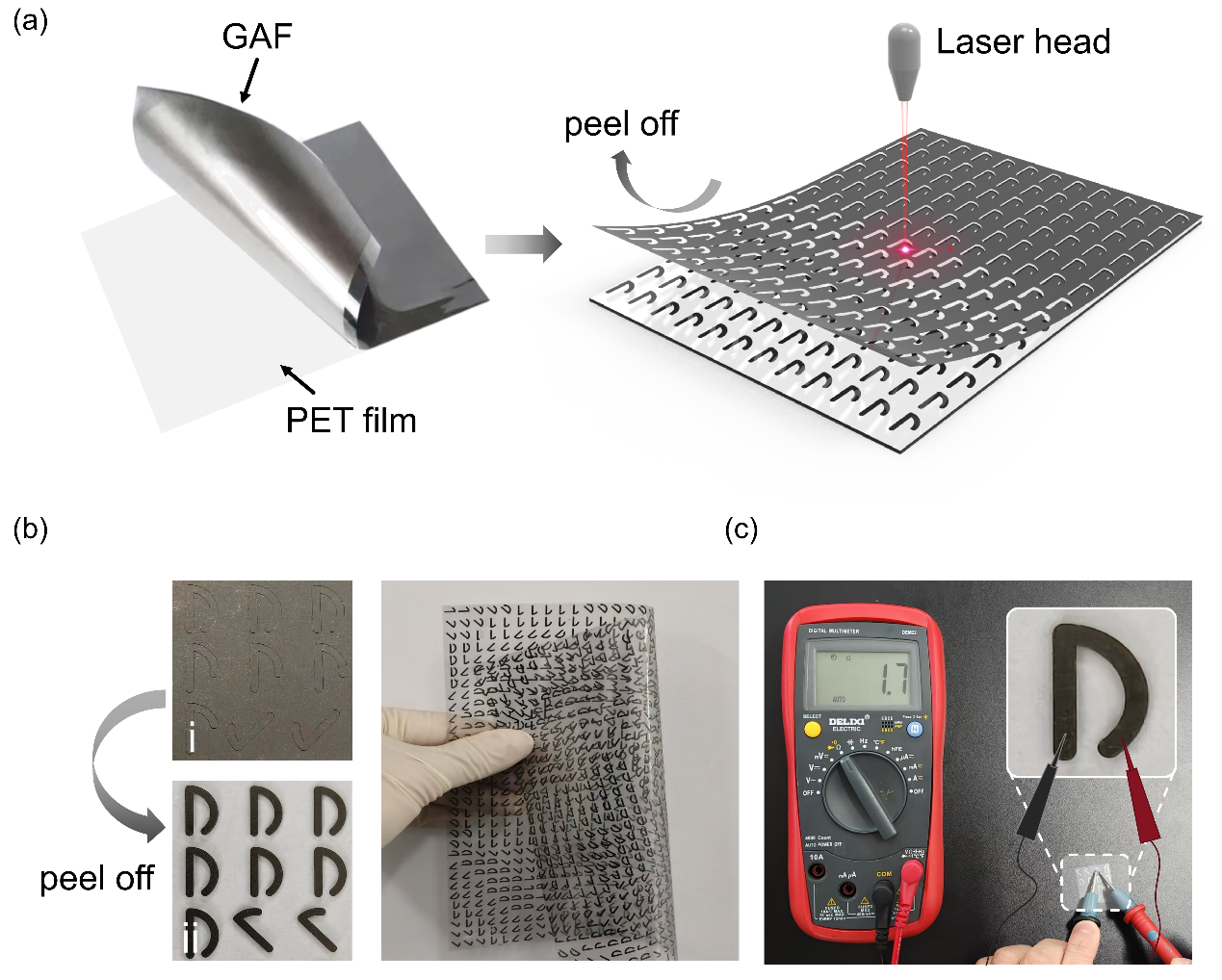


**Figure S7.** (a) Fabrication process of the GAF-based metasurface. (b) Photographs of the GAF film (i) before and (ii) after laser ablation and peeling. (c) measurement of resistance using a digital multimeter.

The specific fabrication process is as follows. A 0.1-mm-thick PET film serves as the flexible substrate, onto which a graphene-assembled film (GAF) is uniformly laminated to form the initial composite structure. As shown in **Figure S7**, a laser ablation process is employed to precisely engrave the designed periodic pattern onto the GAF surface. The laser power is maintained at a low level to ensure clean pattern transfer without damaging the underlying PET substrate. After patterning, excess GAF material is peeled off in a stripping step, leaving well-defined structural boundaries free of residue. Finally, the patterned layers are stacked and bonded using a 1.9-mm-thick PET spacer as the intermediate support, resulting in the final array configuration.

Codebook

A digital multimeter was employed in a two-point contact configuration to measure the resistance across a predefined path with a length (circumference) of approximately 9 mm and a thickness of 0.05 mm. The measured resistance value was 1.7 Ω.

Conductivity (*σ*):

$\sigma=\frac{L}{R\times A}=\frac{9.0\times10^{-3}}{1.7\times\left(5\times10^{-9}\right)}\approx1.06\times10^{6}S/m$ (S4)

A rough measurement of the conductive path using a digital multimeter indicated a bulk conductivity on the order of ~1.06×10^6^ *S/m*.

**Section 6. Quantitative analysis of longitudinal tolerance and spatial locking mechanism**

**
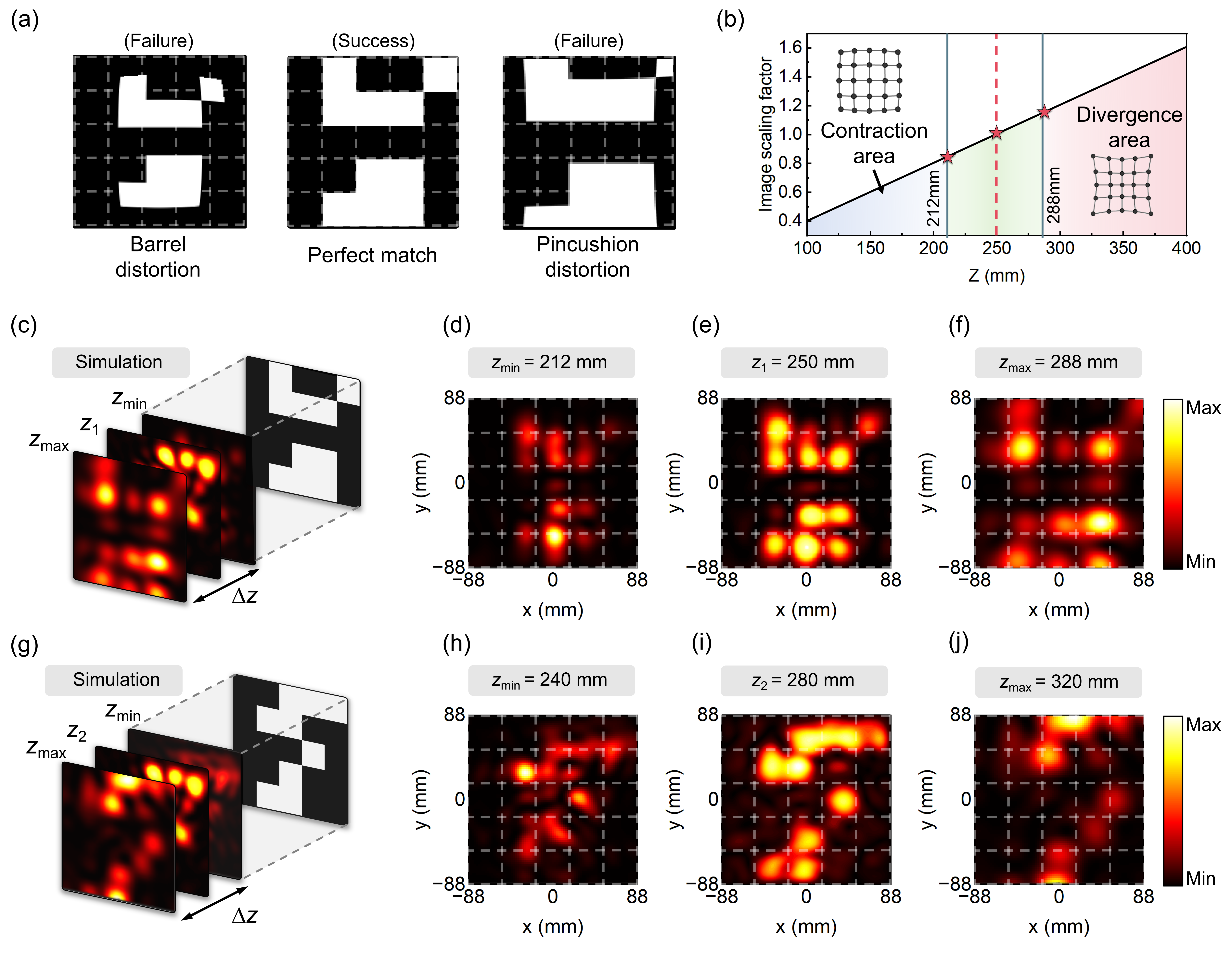
**

**Figure S8**. Quantitative analysis of longitudinal tolerance and holographic pattern scaling. (a) Comparison of three focal scenarios: Barrel distortion (contraction failure), perfect match (success), and pincushion distortion (divergence failure) under varying axial distances. (b) The linear dependence of the geometric scaling factor on the axial distance (*z*) relative to the focal plane. (c) The target ground-truth image for the first channel compared with the experimentally measured amplitude distributions at (d) 212 mm, (e) 250 mm, and (f) 288 mm. (g) The target image for the second channel alongside the corresponding amplitude maps measured at (h) 240 mm, (i) 280 mm, and (j) 320 mm.

The geometric fidelity of the retrieved 5×5 key matrix is fundamentally governed by the diffraction propagation distance *z*. To establish a universal theoretical framework capable of predicting the effective working window across arbitrary target focal planes, we derive the generalized radial displacement function $\Delta r\left(z\right)$. Unlike conventional holographic imaging that targets visual sharpness, our pipeline ultimately depends on stable recovery of a 5 × 5 binary codeword after block-wise energy integration and thresholding. Therefore, a mismatch in reconstruction distance *z* should be interpreted as a decryptability constraint that can trigger discrete bit flips, rather than merely a gradual blurring effect. As schematically illustrated in **Figure S8**, axial defocusing leads to two dominant failure modes, namely contraction and divergence, both of which induce systematic mis-registration between focal spots and the fixed 5 × 5 detector grid. For a signal bit located at a radial distance $r_{0}$ on the design focal plane *z*_0_, its apparent radial position on an arbitrary reconstruction plane at axial distance *z* results from the combined effect of paraxial geometric scaling and defocus-induced diffraction distortion. To make this explicit, we first define the projected position as:

$r\left(z\right)=\frac{z}{z_{0}}r_{0}+D\left(z,r_{0}\right)$ (S5)

where the first term captures the ideal geometric magnification and $D\left(z,r_{0}\right)$ summarizes higher-order distortion contributions arising from non-ideal diffraction and aberration effects. Accordingly, the displacement relative to the fixed detector grid is defined as $\Delta r\left(z\right)=r\left(z\right)-r_{0}$. The strict linear dependence of the geometric scaling factor $z/z_{0}$ is quantified and validated in **Figure S8b**. In addition, by retaining the secondary term $D\left(z,r_{0}\right)$, we can formulate a conservative safety criterion that ensures reliable binary extraction for the 5 × 5 matrix. The physical significance of the parameters in this formulation is directly tied to the 5 × 5 matrix geometry (total width $35.2\text{ }\mathrm{mm}$). Specifically, the maximum radius *r_max_* = 24.89 mm sets the evaluation boundary, while the spatial tolerance *τ* = 3.52 mm (half of a grid cell width) specifies the maximum allowable positional error for block-wise binarization. Therefore, the effective axial decoding window is defined by the set of axial positions *z* satisfying:

$\Delta r_{\max}\left(z\right)=\left|r\left(z\right)-r_{0}\right|_{r_{0}=r_{0}}\leq\tau$ (S6)

which explicitly links axial defocus to discrete decoding failure through the grid-based decision rule. Subsequently, the simulation of the reconstructed field amplitude distributions graphically validates the theoretical boundaries derived from the scaling inequality. For the first channel, where the target ground-truth is shown in **Figure S8c**, the amplitude maps at three critical positions clearly demonstrate the mechanism of failure. At *z* = 212 mm (**Figure S8d**), a position slightly beyond the near-field scaling limit, the pattern exhibits distinct inward collapse, where the peripheral focal spots shift radially inward, resulting in their amplitude distributions bleeding into the adjacent, incorrect grid cells and triggering recognition error (Contraction Failure). Conversely, at the far-field limit of *z* = 288 mm (**Figure S8f**), the pattern undergoes significant expansion, causing the outermost signal blocks to be pushed toward and often beyond the physical boundaries of the fixed detector area (Divergence Failure). Optimal alignment, characterized by spatially isolated and centered focal spots, is strictly confined to the design focal plane at *z* = 250 mm (**Figure S8e**). A consistent evolution of alignment errors is observed for the second channel (Target in **Figure S8g**), as evidenced by the amplitude measurements at *z* = 240 mm (**Figure S8h**), *z* = 280 mm (**Figure S8i**), and *z* = 320 mm (**Figure S8j**). These images collectively confirm that the integrity of the binary key matrix is highly sensitive to axial position, demonstrating how even small deviations from the ideal focal depth swiftly compromise the amplitude thresholding and grid statistics algorithms due to excessive pixel crosstalk and positional displacement.

**Section 7. Multi frequency performance**


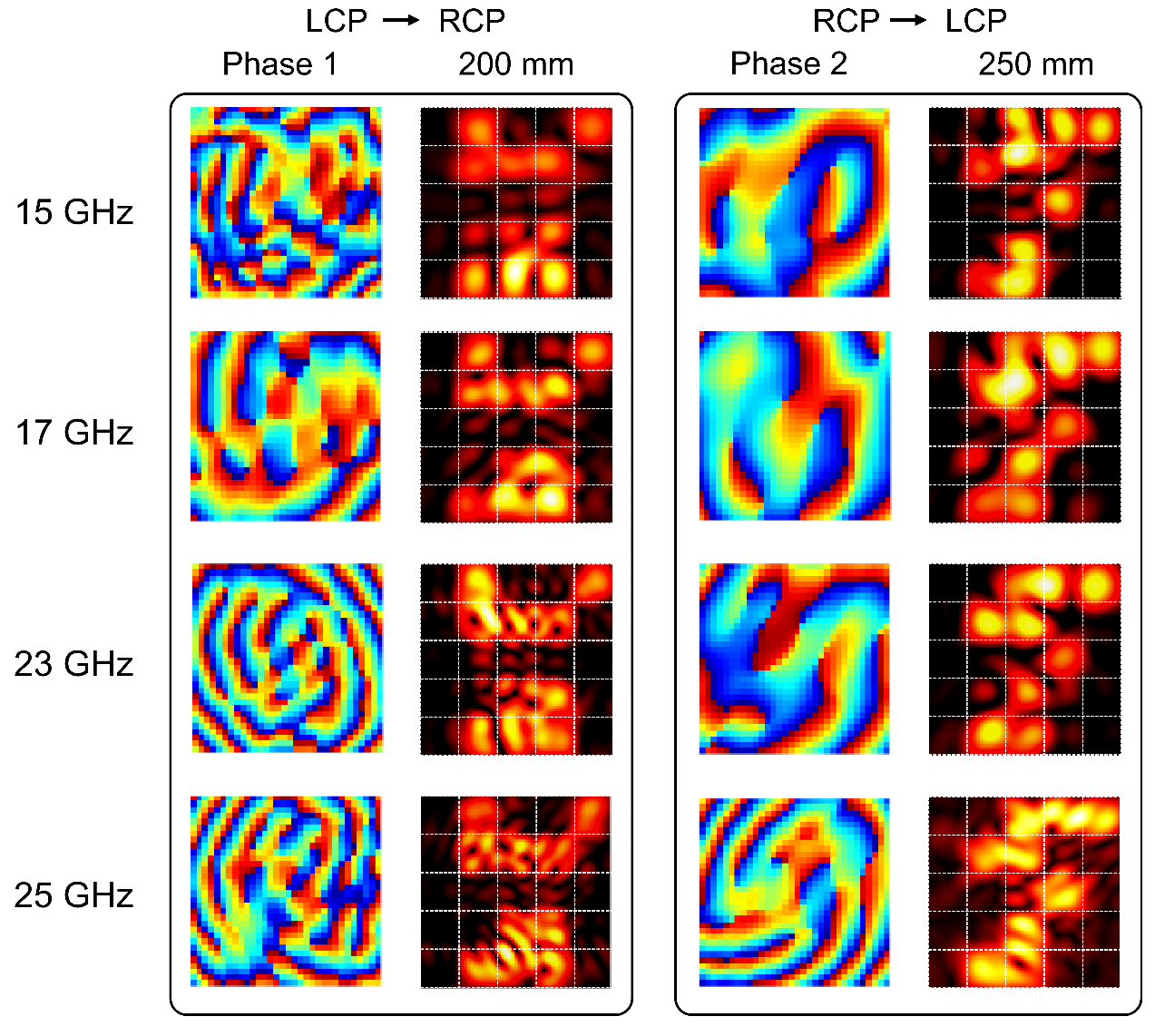


**Figure S9.** Broadband Performance Validation: Recovered Holographic Phase Distributions and Simulated Reconstruction.

**Figure S9** presents the recovered discrete holographic phase distributions for the LCP and RCP channels at four representative frequencies (15 GHz, 17 GHz, 23 GHz, and 25 GHz), which were calculated using the GS algorithm to project the target key images. Based on these phase distributions, full-wave simulations of the holographic reconstructions were performed. The results demonstrate that, despite a shift in the operational frequency, the metasurface can still reconstruct the core pattern of the holographic keys, albeit with potential variations in focus quality, spot size, and background noise. These variations arise primarily from the frequency-dependent phase dispersion of the unit cells. However, this inherent physical limitation is effectively mitigated by the system's block-based preprocessing strategy. By dividing the holographic image into discrete pixel blocks with uniform intensity values, the encryption scheme ensures that minor intensity fluctuations within individual blocks do not significantly compromise the overall recognizability of the reconstructed keys. This approach provides the system with remarkable noise resilience while maintaining the security protocol's integrity across the operational bandwidth.

**Section 8. Visualization of the dynamic codebook library.**


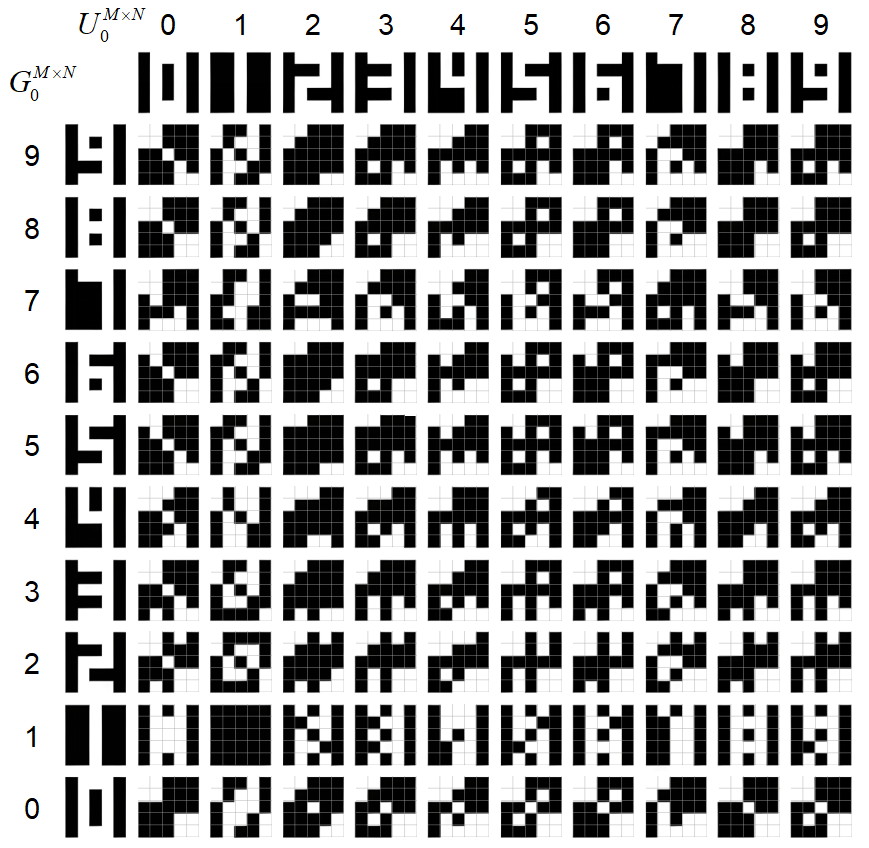


**Figure S10.** Dynamic Codebook $T_{c}^{M\times N}$ library. The matrix displays the specific 5×5 binary pixel distributions required to strictly map the intermediate decoy states ($U_{0}^{M\times N}$) to the original information ($G_{0}^{M\times N}$) via the logical XOR operation.

To ensure the high-fidelity retrieval of the secret information, the Dynamic Codebook ($T_{c}^{M\times N}$) serves as the core of the cyber-decryption stage. Unlike static filters, the codebook acts as a content-dependent library of keys. **Figure S10** illustrates the generation logic of this codebook for a numerical application scenario (digits 0–9). The horizontal axis represents the visual patterns of the physical decoy ($U_{0}^{M\times N}$), while the vertical axis represents the patterns of the intended original image ($G_{0}^{M\times N}$).

**Section 9.** **Evaluation of the threshold and demonstration of capacity scalability**

**
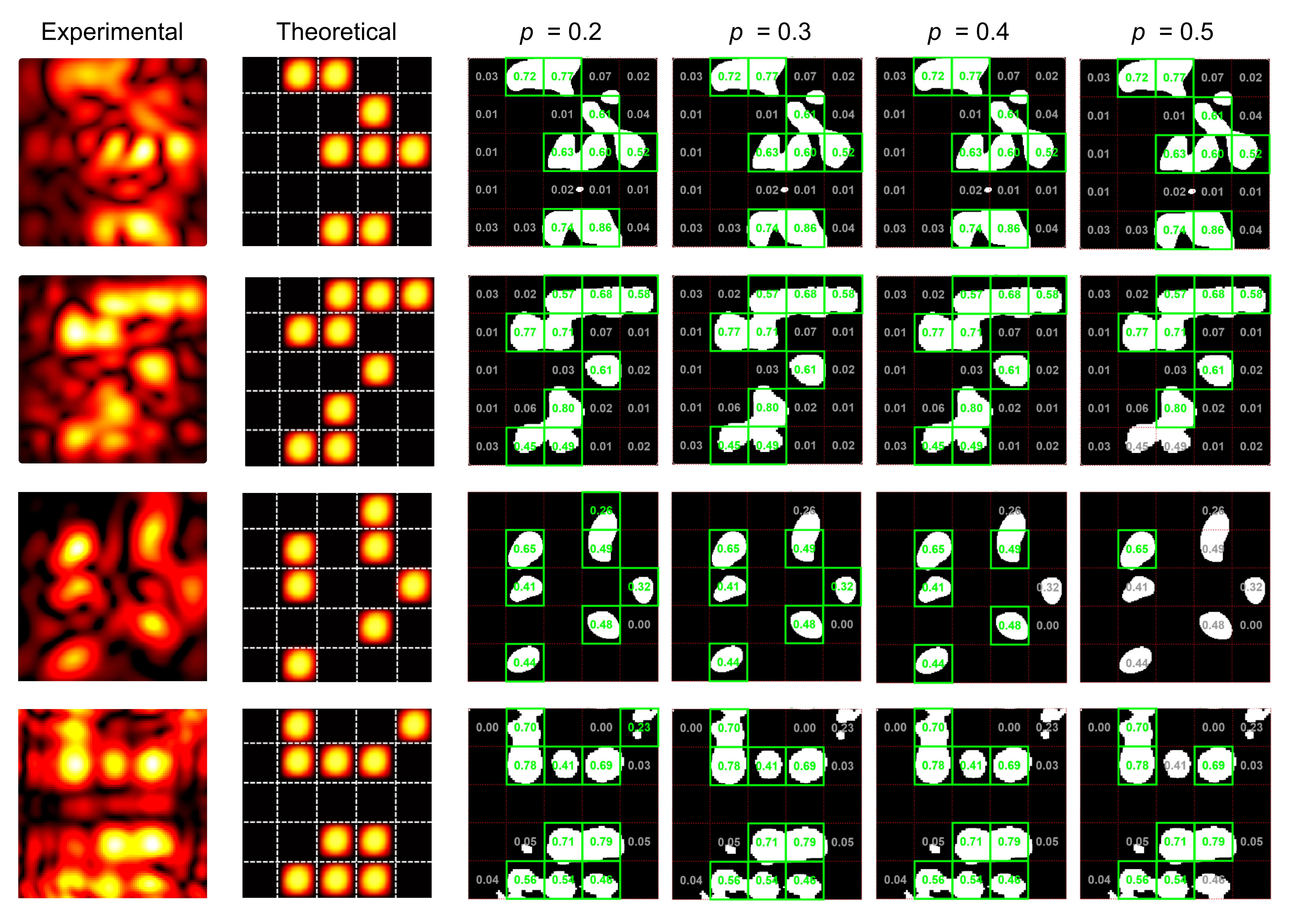
**

**Figure S11.** Experimental holographic images, theoretical ground-truth patterns, and matrix extraction results under varying thresholds.

**Figure S11** evaluates the parameter selection for Eqn. (10). Physical speckle noise fragments reconstructed holographic spots. Valid logical blocks in experimental data exhibit active pixel ratios dropping to 0.23 and 0.26. Thresholds *p* ≥ 0.3 erroneously discard these signals, leading to false negatives. Background noise ratios are strictly suppressed below 0.07 by Otsu binarization. Consequently, *p* = 0.2 provides a robust morphological boundary, tolerating severe physical signal degradation while preventing bit errors.

**
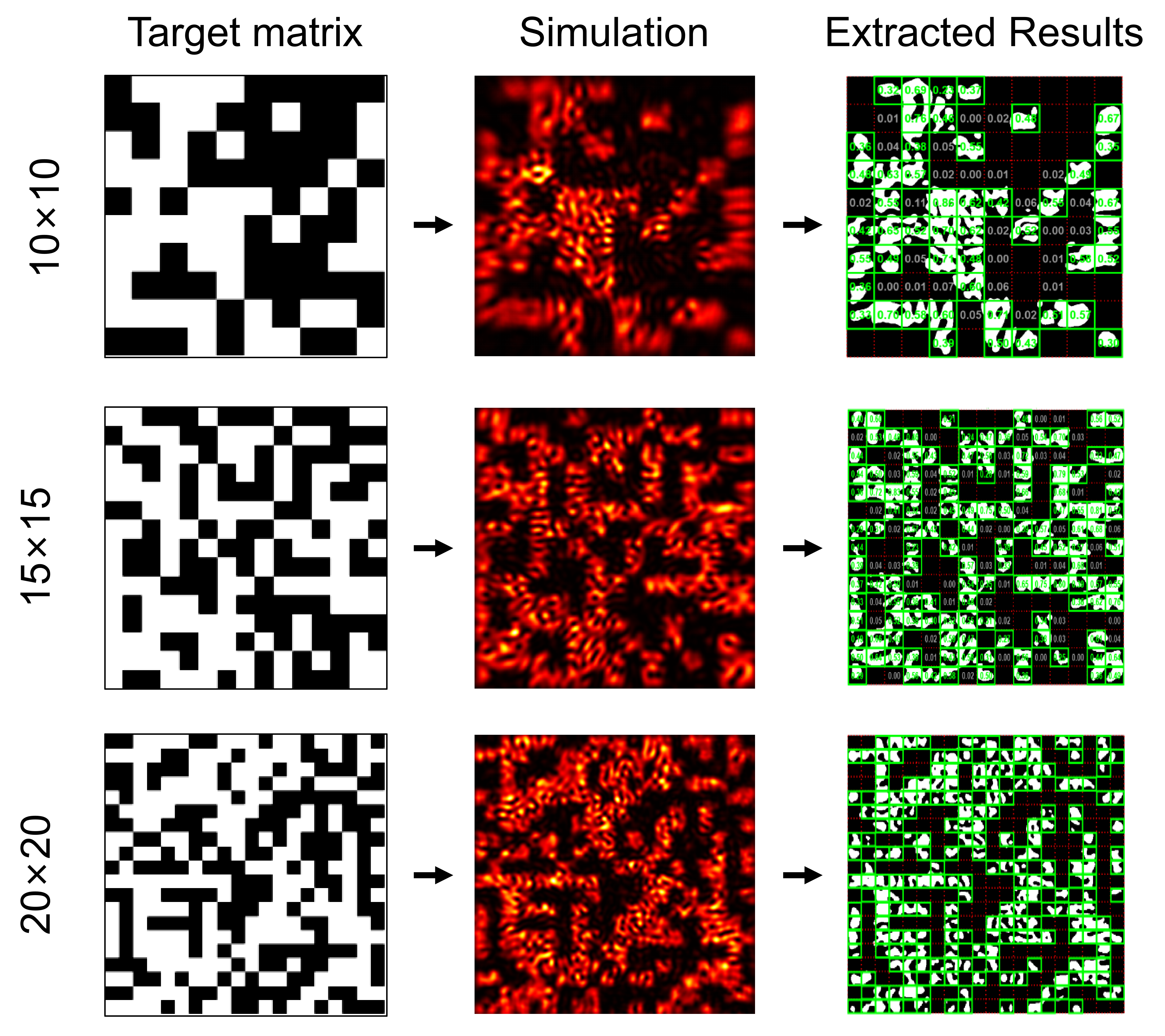
**

**Figure S12.** Extraction performance on expanded matrix capacities.

To demonstrate capacity scalability, we tested information matrices of 10×10, 15×15, and 20×20 (**Figure S12**). The algorithm reliably extracts the 100-bit and 225-bit matrices with 0 BER. For the 20×20 matrix, despite dense speckle interference, it maintains robust extraction performance. Governed by the scale invariance of Maxwell's equations, this architecture is, in principle, scalable to higher frequencies through structural miniaturization, provided that suitable fabrication accuracy and material responses are maintained.


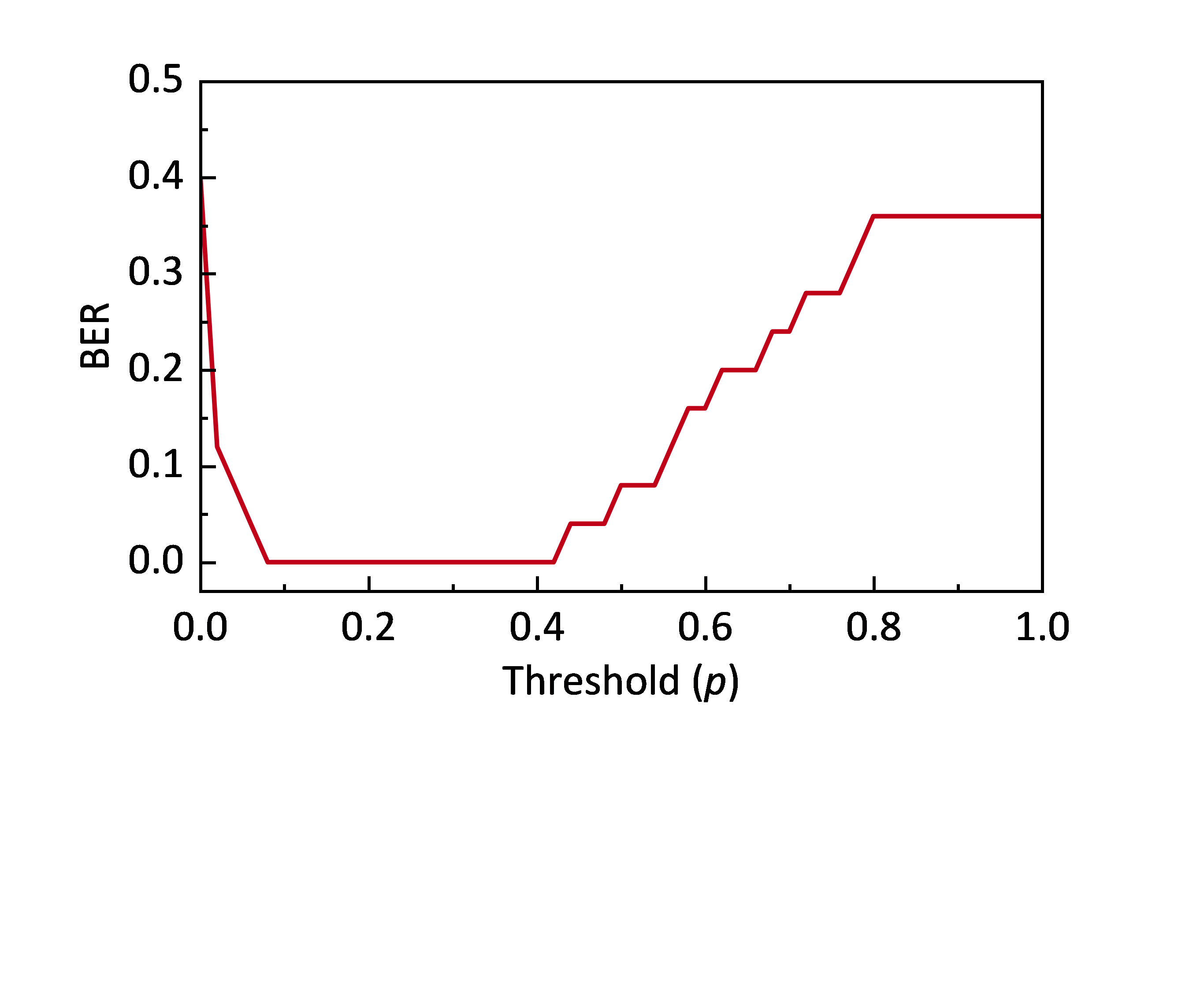


**Figure S13.** Evaluation of the extraction BER as a function of the morphological threshold (*p*).

Figure S13 plots the BER of the extracted matrix against the morphological threshold (*p*). The results reveal a broad, error-free window spanning from *p* = 0.08 to 0.42. Thresholds below 0.08 fail to suppress residual background noise, whereas thresholds above 0.42 discard valid but fragmented signal pixels, both resulting in elevated extraction errors. This quantitative analysis verifies that the selected threshold of *p* = 0.2 operates safely within the robust margin, ensuring accurate matrix recovery.
